# Study of Combinatorial Drug Synergy of Novel Acridone Derivatives With Temozolomide Using *in-silico* and *in-vitro* Methods in the Treatment of Drug-Resistant Glioma

**DOI:** 10.3389/fonc.2021.625899

**Published:** 2021-03-15

**Authors:** Malobika Chakravarty, Piyali Ganguli, Manikanta Murahari, Ram Rup Sarkar, Godefridus Johannes Peters, Y. C. Mayur

**Affiliations:** ^1^Department of Pharmaceutical Chemistry, Shobhaben Pratapbhai Patel School of Pharmacy and Technology Management, SVKM's NMIMS, Mumbai, India; ^2^Chemical Engineering and Process Development Division, CSIR-National Chemical Laboratory, Pune, India; ^3^Academy of Scientific and Innovative Research (AcSIR), Ghaziabad, India; ^4^Department of Pharmaceutical Chemistry, Faculty of Pharmacy, M.S. Ramaiah University of Applied Sciences, Bengaluru, India; ^5^Department of Biochemistry, Medical University of Gdansk, Gdansk, Poland; ^6^Laboratory Medical Oncology, Amsterdam University Medical Centers, Location VUMC, Amsterdam, Netherlands

**Keywords:** acridone derivatives, drug combinations, synergy index, mathematical model, Glioma, drug resistance

## Abstract

Drug resistance is one of the critical challenges faced in the treatment of Glioma. There are only limited drugs available in the treatment of Glioma and among them Temozolomide (TMZ) has shown some effectiveness in treating Glioma patients, however, the rate of recovery remains poor due to the inability of this drug to act on the drug resistant tumor sub-populations. Hence, in this study three novel Acridone derivative drugs AC2, AC7, and AC26 have been proposed. These molecules when combined with TMZ show major tumor cytotoxicity that is effective in suppressing growth of cancer cells in both drug sensitive and resistant sub-populations of a tumor. In this study a novel mathematical model has been developed to explore the various drug combinations that may be useful for the treatment of resistant Glioma and show that the combinations of TMZ and Acridone derivatives have a synergistic effect. Also, acute toxicity studies of all three acridone derivatives were carried out for 14 days and were found safe for oral administration of 400 mg/kg body weight on albino Wistar rats. Molecular Docking studies of acridone derivatives with P-glycoprotein (P-gp), multiple resistant protein (MRP), and O6-methylguanine-DNA methyltransferase (MGMT) revealed different binding affinities to the transporters contributing to drug resistance. It is observed that while the Acridone derivatives bind with these drug resistance causing proteins, the TMZ can produce its cytotoxicity at a much lower concentration leading to the synergistic effect. The *in silico* analysis corroborate well with our experimental findings using TMZ resistant (T-98) and drug sensitive (U-87) Glioma cell lines and we propose three novel drug combinations (TMZ with AC2, AC7, and AC26) and dosages that show high synergy, high selectivity and low collateral toxicity for the use in the treatment of drug resistant Glioma, which could be future drugs in the treatment of Glioblastoma.

## Introduction

Glioblastoma multiforme or Gliomas are the most commonly occurring primary tumors in the brain and spinal cord. Repeated failures and multiple challenges are seen in treating Glioma due to the development of drug resistance, recurrence, collateral toxicity to healthy cells, and detrimental adverse effects. Amongst these, one of the greatest challenges faced is the development of drug resistance, which can occur as a result of several complex mechanisms. These include poor absorption of the drug, efflux transport pumps, metabolic reprogramming, de-regulation in gene, and protein expression responsible for apoptosis as well as tumor heterogeneity (1–3).

**Figure 1:**
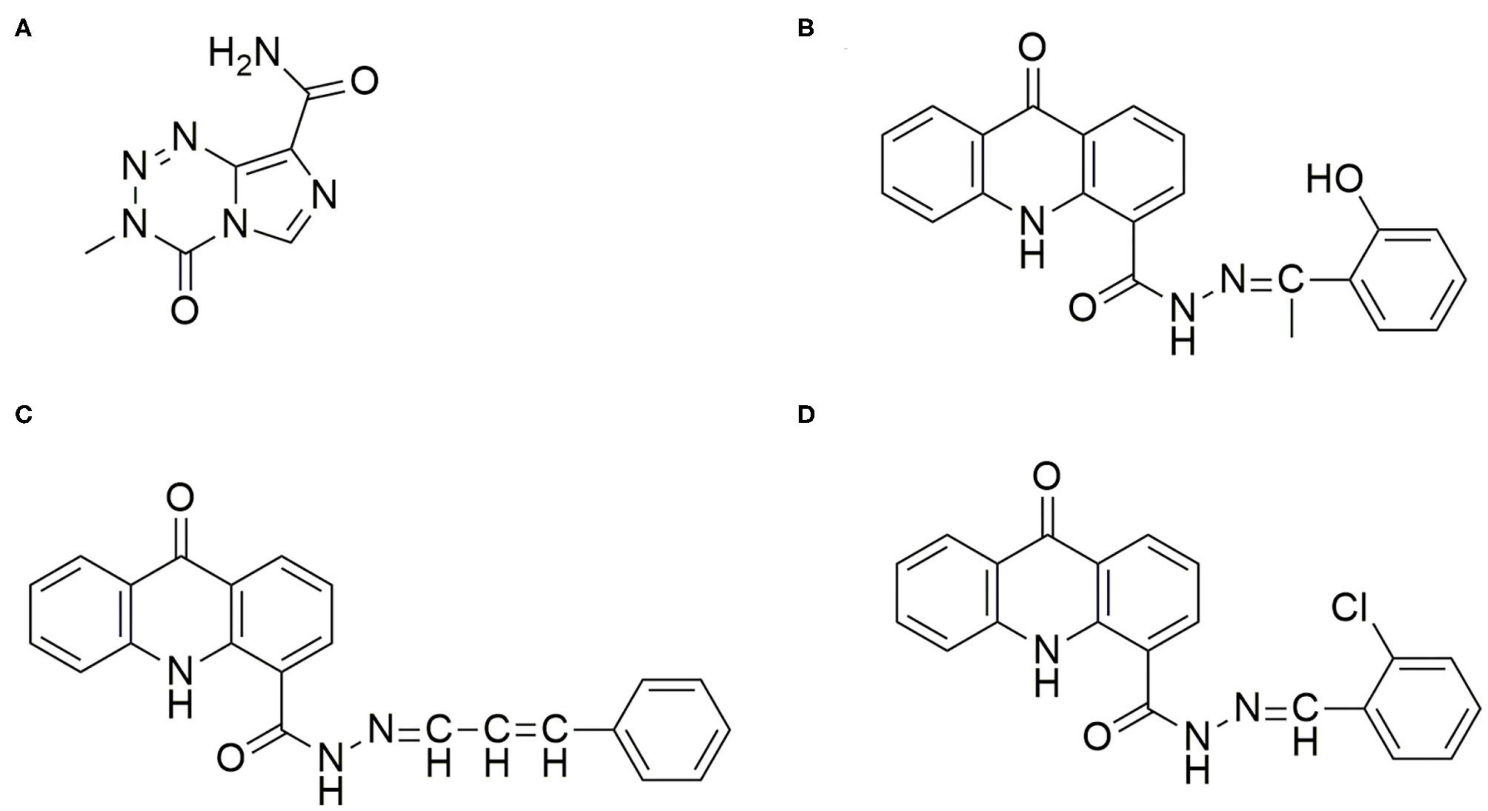
Chemical Structure of the drugs TMZ and Acridone derivatives for Glioma Therapy. **(A)** Temozolomide (TMZ): (3-Methyl-4-oxo-3,4-dihydroimidazo[5,1-d][1,2,3,5]tetrazine-8-carboxamide; **(B)** AC26: N'(1-(2-hydroxyphenyl)ethylidene)-9-oxo-9,10-dihydroacridine-4-carbohydrazide; **(C)** AC2: 9-oxo-N'-(3-phenylallylidene)-9,10-dihydroacridine-4-carbohydrazide; **(D)** AC7: N'-(2-chlorobenzylidene)-9-oxo-9,10-dihydroacridine-4-carbohydrazide.

Over the past few decades, several chemotherapeutic drugs like Carmustine, Lomustine, Vincristine, Cisplatin, Bevacizumab etc., have been studied for the treatment of Glioma (4, 5). However, at present, Temozolomide (TMZ) is the one of the well-known and the most effective drug used for the treatment and management of Glioma. It is an alkylating agent belonging to the tetrazine class (Figure 1A) having a molecular weight of 194 g/mol and has shown the ability to penetrate the Blood Brain Barrier (BBB) (6). Reports suggest TMZ increases survival advantage by 2.5 times when the drug is administered along with surgery and radiotherapy (7). It is an alkylating agent that transfers a methyl group (CH_3_) to a purine base of DNA (N7-guanine, O6-guanine, and N3-adenine) causing both single and double stranded breaks leading to apoptotic cell death (8). However, it has been observed that although the malignant cells respond to this TMZ induced apoptosis during the initial phases, they gradually develop resistance to it during the later phase of cancer progression. This is because the cytotoxic action of TMZ is reversed by removal of methyl group from O6-methylguanine (O6-MeG) by the methylguanine methyltransferase enzyme (MGMT) (9). The MGMT is an enzyme that is overexpressed in the tumor and the most common reason for the development of drug resistance. Additionally, administration of TMZ results in certain side effects like alopecia, fatigue, nausea, vomiting, headache, constipation, anorexia, convulsions, rash, fever, dizziness, amnesia, insomnia, lymphopenia, thrombocytopenia, neutropenia, and leucopenia, and also leads to severe toxicity at high doses (more than 250 μM) (10). Furthermore, as per equation proposed by Levin-log permeability it was observed that TMZ has a very low brain capillary permeability with a value close to 2.7 × 10^−6^ cm/s (11). Hence, it may be assumed that permeability of TMZ can be improved by combining it with another lipophilic drug and these acridone molecules fulfill this gap.

However, regardless of these obstacles, TMZ is used as a standard drug in treating Glioma and is the most promising drug known to treat Glioma till today. Hence, in this present study we aim to thwart resistance by combining TMZ with acridone molecules which may have a synergistic effect and help in effectively treating the drug resistant tumor cells. Nevertheless, the question arises which other drugs should be selected that would give synergistic effect with TMZ? In order to circumvent the shortcomings faced due to the use of the known drugs, it has become crucial to consider novel drugs for fighting Glioma and the drug resistance. In one of our recent work, we have shown that certain novel acridone derivatives have shown high cytotoxic effect against Cervical, Lung, and Breast Cancers (12, 13). In Breast Cancer studies, using MCF-7 cell lines, it has been observed that certain acridone derivatives have the ability to bind to multiple targets and prevent multiple mechanisms responsible for drug resistance in cancer. These molecules can intercalate with DNA and inhibit the process of cell division, initiate reactive oxygen species (ROS) mediated oxidative stress, and bind to the proteins expressed in the plasma membrane responsible for efflux of the drugs like P-glycoprotein(P-gp) (14). The studies have revealed that overexpression of proteins and actions of efflux pumps are the main reason for the drug resistance as this leads to drugs failing to accumulate and exert their activity at the site of action. Apart from this, acridone moieties also showed unique properties of high lipophilicity enabling penetration of the drug into the BBB. It has also been observed that certain structural modifications in these acridone derivatives could result into more potent drugs AC2, AC7, and AC26 which have shown 100 times more cytotoxicity in comparison to the other acridone derivatives (15) (Figures 1B–D). These Acridone compounds (AC2, AC7, and AC26) on evaluation of histopathology demonstrated liver with minimal hyperchromatic, anaplasia, and cellular infiltration. Studies conducted using these compounds showed no cardiotoxicity, nephrotoxicity and necrosis. In recent past, these acridone molecules were able to successfully act on breast cancer MCF-7 cell line (Michigan Cancer Foundation-7) at lower concentration and have shown ability to modulate cytotoxicity in drug resistant MCF-7/ADR (Adriamycin) cell lines when administered in combination with Vinblastine (14). Thus, due to high cytotoxicity of acridone compounds at low dose in MCF-7/ADR cell lines and because of their ability to overcome drug resistance, we aim to test its effectiveness in the treatment of Glioma. In order to have an effective Glioma therapy with minimal toxicity and to overcome drug resistance, in this study, we have tested different combinations of acridone derivatives with TMZ. Here, for the first time *in-vitro* and *in-silico* strategies have been employed together to evaluate efficacy of combinatorial drugs-TMZ + AC26, TMZ + AC2, and TMZ + AC7 in the treatment of Glioma and to evaluate their synergistic action to overcome the drug resistance in Glioma.

Sulforhodamine B (SRB) Assay of the novel acridone derivatives, *viz*., AC2, AC7, and AC26 have been performed on U-87 (Uppsala 87-WT) and T-98 (Temozolomide resistant) malignant Glioma cell lines to determine the effectiveness of the individual drugs on the drug sensitive and drug resistant cancer cells. In order to gain insights into the molecular mechanism underlying the effectiveness of the Acridone derivatives in overcoming the drug resistance in Glioma, Molecular Docking studies have been performed to compare the binding affinities of the three Acridone derivatives with MGMT, P-gp, and MRP proteins which are responsible for conferring resistance to the Glioma cells. Thereafter, to determine the effectiveness of these drugs in combination with TMZ and to observe the dose responses under different dosage combinations, we have developed a mathematical model to mimic the effect of these drugs on heterogeneous subpopulation of cancer cells (drug sensitive cancer cells and drug resistant cancer cells) in a tumor. The model has been parameterized using the experimental data and its outcome have been validated with our *in vitro* experiments on the Glioma cell lines in order to ensure correct predictions and to provide the optimum concentration of both the drugs within the toxicity limits for maximum efficacy. The dose response matrices generated from the simulation of the mathematical model have been used for screening 10,000 combinations of doses for each pair of drugs for evaluating the synergistic intensity of each dose combinations of the TMZ and Acridone derivative using *in-silico* method. Experimental studies have also been performed to validate the synergistic dosage combinations of each pair of drugs. This study throws light on new treatment strategies for Glioma by the selection of most beneficial doses of the combinatorial drugs with minimum side effects and determination of the optimum doses for synergy and highest efficacy.

## Materials and Methods

### Drugs Characterization

Characterization of acridone derivatives AC2, AC7, and AC26 were carried out by using all the chemicals of analytical grade. Hydrochloric acid and sodium hydroxide were procured from Sisco Research Lab Pvt. Ltd (Mumbai, India). Potassium dihydrogen orthophosphate, methanol and ortho-phosphoric acid (85% pure) was received from Loba Chemie Pvt. Ltd. (Mumbai, India). Sodium chloride was received from SD Fine-Chem Limited (Mumbai, India). Milli-pore water was used throughout the study. Chemical information like the molecular weight of compounds was determined by obtaining ESI-MS spectroscopy using methanol as solvent. pH calculated by using pH analyzer (Lab India) and PKa determined by using UV-Vis spectrophotometer (Shimadzu, Japan). Melting point was calculated by Digital Melting point apparatus (Veego India). Lipophilicity was determined by using software like Chem Draw 15 and ALGOPS 2.1 (16). In order to determine the Blood Brain Barrier (BBB) permeability of the compounds, we have used Online BBB Predictor (https://www.cbligand.org/BBB/predictor.php) using SVM Machine Learning algorithm and PubChem as the fingerprint (17).

### Molecular Docking

Molecular docking of the Acridone derivatives AC2, AC7, and AC26 were performed to investigate the binding interactions and patterns at the active pockets of the drug resistance causing target proteins. X-ray crystallographic structures of the target proteins [i.e., P-gp (PDB ID- 6QEX), MRP (PDB ID- 2CBZ), and MGMT (PDB ID- 1QNT) were retrieved from Protein Data Bank (PDB) (18)]. All the crystallographic structures were processed individually in Discovery Studio Visualizer. All the water molecules, unwanted chains, heteroatoms of respective protein structure were removed and hydrogen atoms were added in Discovery Studio Visualizer (19). Receptor cavities of individual proteins were identified by selecting the co-crystallized ligand in the Discovery Studio Visualizer and attributes were documented for docking calculations (19). Thereafter the processed proteins were loaded to AutoDock Tools, both Kollman and Gasteiger charges were added and saved in pdbqt format (20). All the ligands were sketched in Avogadro software and optimized with Universal Force Field (UFF) and Steepest Descent algorithm (21). Prepared ligands were loaded to AutoDock and saved in pdbqt format. Attributes of individual target protein obtained from Discovery Studio (Supplementary Table 1), name of protein and ligands were written in configuration file and submitted to molecular docking. Docking parameters were optimized using Genetic Algorithm (GA) approach with 1,000 runs. Docked pose with highest binding affinity was visualized and interaction analysis was performed in Biovia Discovery Studio Visualizer. All the docking calculations were carried out in a laptop running with Windows 10 64 bit operating system, 8GB RAM and i3 processor.

### Sulforhodamine B Assay for Individual Drugs

In order to test the growth inhibition potential of the TMZ and Acridone derivatives on Glioma, the U-87 (Uppsala 87) Glioma cell lines and T-98 (Temozolomide resistant) Glioma cell lines were treated with Temozolomide (TMZ) and Acridone derivatives AC2, AC7, and AC26. The African Green Monkey Kidney Vero cell lines were used as control for the study. The Vero cell lines were obtained from the National Center for Cell Sciences (NCCS), Pune, while the U-87 and T-98 cell lines were carried in the lab of Dr. GJ Peters, Cancer Center Amsterdam (CCA), VU University Medical Center, Amsterdam, Netherlands. The Mycoplasma testing was done by using the Universal Mycoplasma Detection Kit (ATCC® 30-1012K™) every 6 months. The Vero cell lines have been used as control as they are normal kidney epithelial cells and are non-cancerous in nature. The Vero cell lines have been routinely used for testing cytotoxicity of small molecules by various researchers (22, 23). Also, in our previous study, Vero cell lines have been used for testing safety and efficacy of Acridone derivatives for Lung, Cervical, and Breast Cancer studies (13).

Cell viability was found using the Sulforhodamine B (SRB) assay to measure the cellular protein content which provides better sensitivity in comparison to MTT assay (24). Moreover, as this method is dependent on the property of the SRB dye, it acts by binding to proteins under slightly acidic conditions and can be exposed to basic conditions for its extraction. The resulting amount of bound dye is then utilized as a proxy for cell mass (25). This cell mass can then be extrapolated to measure cell growth.

Glioma cells were cultured in Gibco (RPMI 1640) complemented with 10% fetal calf serum (Gibco). The cultures were then treated with trypsin-EDTA in order to detach/separate the cells from their culture flasks. The quickly proliferating cells were harvested, counted and plated at suitable concentrations in 96-well microplates. These microplates were subsequently incubated for 24 h. After incubation, drug compounds were dissolved in the culture media and placed in 96 well plates in triplicates, which were again incubated at 37°C under 5% CO_2_ for 72 h. 72 h later, the plates were removed and the cells were treated with cold Trichloroacetic acid (TCA) to fix the cultures and. 0.4% of SRB dissolved in 1% acetic acid was then added to the culture in order to stain the cells. Next, the bound stain was dissolved in 150 μl of 10 mM unbuffered Tris base left on a gyrator shaker. Thereafter, absorbance of the solution was measured at 540 nm utilizing a microplate reader. Absorbance readings (triplicate values) recorded were used to calculate percentage growth inhibition due to the effect of drugs using the following equations.

% Cell growth = Absorbance sample/Absorbance of control or untreated × 100

% Growth inhibition = 100 - % cell growth

The Inhibitory concentration (IC_50_) of the drugs were determined on the basis of concentration that induced 50% growth inhibition of the treated cells in comparison to untreated cells after 72-h treatment (as given in **Table 4**). The IC_50_ was calculated based on the log graph sheet which was developed in-house at the CCA, VU University Medical Center.

#### Selectivity Index

Selectivity Index (SI) is a ratio that measures the window between cytotoxicity and anticancer activity of the drugs (26). Thus, to evaluate effectiveness of all the 4 drugs, SI has been calculated using the formula SI = CC_50_ of Vero cell line /IC_50_ of Cancer cell line, after 72 h of TMZ/AC treatment (27). Here, IC_50_ is the inhibition concentration for inhibiting 50% of cancer cells and CC_50_ is cytotoxic concentration causing death of 50% viable cells in the host. Ideally, IC_50_ concentration should be below CC_50_ concentration suggesting that cancer cells are killed before host cells.

### Model Development

The data obtained from the *in-vitro* experiments were used to develop an Ordinary Differential Equations (ODEs) based model that can mimic the tumor development *in-silico*. The model consists of 4 variables representing the heterogeneous sub-populations of cells in a tumor and 29 parameters that govern the differentiation pattern and growth dynamics of each sub-population. The model has been calibrated and parameters set to capture the growth dynamics of U-87 and T-98 cells that represent the drug resistant and drug sensitive cell sub-populations in a developing tumor. The primary objective of this model development is to screen the effect of varying dosage combinations of TMZ and the Acridone derivatives on the tumor growth to determine the dosage combinations showing high synergistic effects within the toxicity limits.

#### Tumor Growth Model

**Figure 2:**
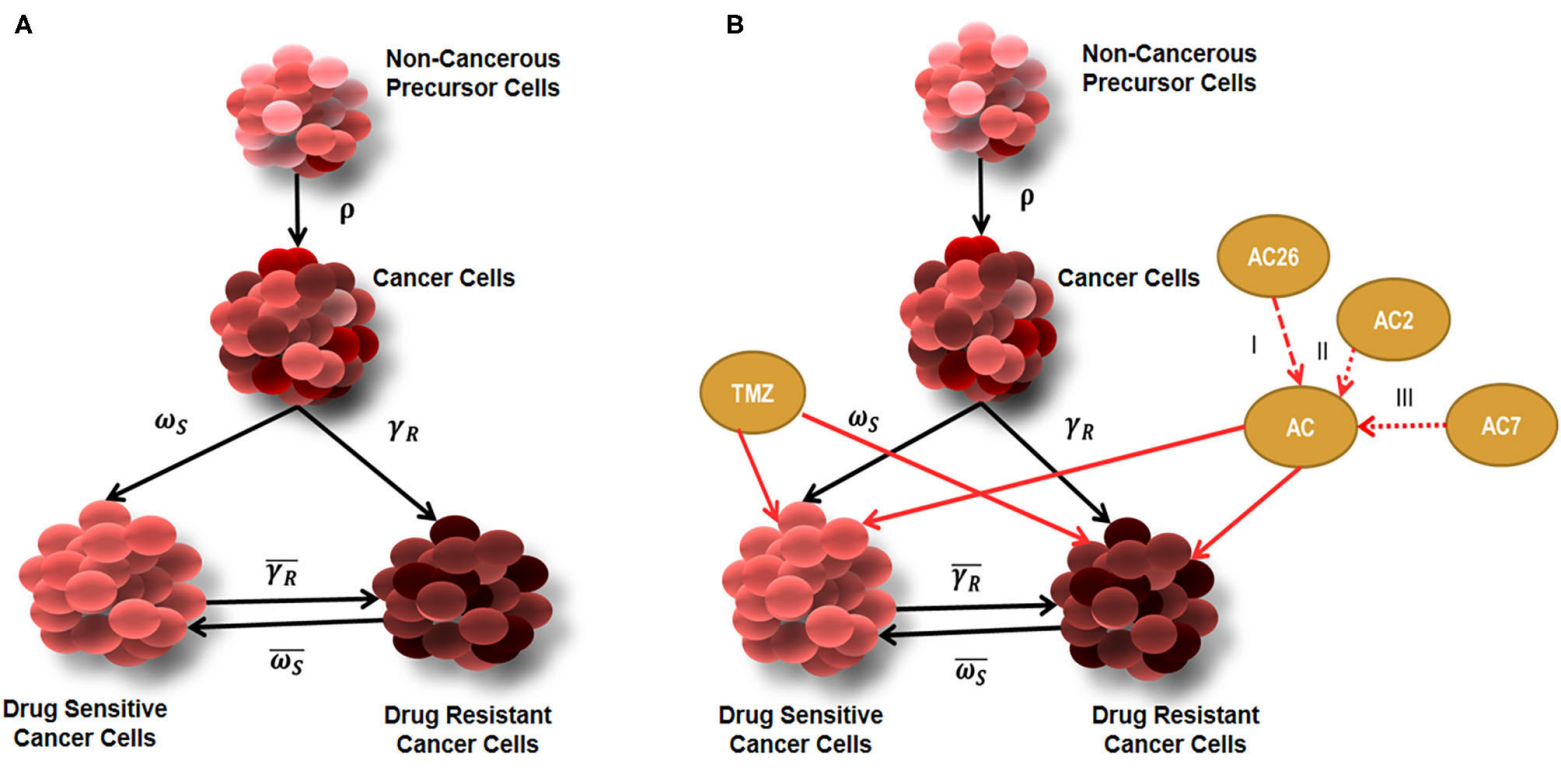
Diagrammatic representation of tumor growth model. **(A)** Tumor growth model showing Non-cancerous precursor cells (N), Cancer cells (C), Drug sensitive (C_S_), and Drug Resistant (C_R_) cells without the effect of drugs; **(B)** Tumor growth model with combinatorial drug therapy using TMZ and Acridone derivatives AC26, AC2, and AC7.

The tumor growth model developed (Figure 2), assumes existence of few non-cancerous precursor cells (N) which tend to acquire mutation (ρ) over a period of time to form cancerous cells (C) (**Equations 1–4**). The process of acquisition of mutation in non-cancerous precursor cell population can be the result of their exposure to several factors like radiations, air pollution, chemicals, other factors (such as Viruses,) etc., (28). Since non-cancerous cells are acquiring mutation with a probability ρ to form cancer cells, the non-cancerous precursor cell population tend to renew their pool of cells with (1- ρ) probability (**Equation 1**). The model further considers that cancer cell population undergoes differentiation to give rise to heterogeneous subpopulations consisting of the resistant cancer cells (C_R_) and sensitive cancer cells (C_S_) (29). The sensitive cells are susceptible to therapy, while therapy is hardly effective against resistant cells. The probability that cancer population would differentiate into Drug resistant cancer population (C_R_) is γ_*R*_ and into Drug sensitive cancer (C_S_) population is ω_*S*_ where γ_*R*_ + ω_*S*_ = 1. Tracing the past footprints in the literature, it can be understood that transitions are allowed to occur between drug sensitive cancer cells and drug resistant cancer cells with more probability $\left({\tilde{\gamma }}_{R}\right)$ of transformation occurring in forward direction and less probability $\left({\tilde{\omega }}_{S}\right)$, that resistant cell population will switch back into sensitive cancer cell population (29, 30). This rare transformation process is well-documented in literature where experiments show that drug resistant cell population reduces in drug free medium (31) or refractoriness to drug therapy can be sometimes reversed by epigenetic programming (32). In mathematical terms, it may be noted here that α_*C*_ and δ_*c*_ denote the natural birth and death rates of C cells. An identical nomenclature has been followed for other types of cells. The resistant cancer cells are developed from the conversion of a C to C_R_. The C_R_ trail an identical pattern of self-renewal and differentiation resulting in the replenishment of the C_R_ pool and development of Cs.

Though a plethora of models exist on the basis of the assumption that proliferation of a constant fraction of tumor volume follows exponential growth but in this model we have considered the widely accepted and well-known model of Gomphertz to describe growth dynamics of cancer cells and their heterogeneous subpopulations (33, 34). Mathematical representation of growth kinetics followed by cancer cells can be given as α_C_*C$*\text{log }\frac{K}{C+\mu }$, where K denotes carrying capacity of the cancerous cells (35). In this model, it has been considered that C_R_ and C_S_ follow identical Gompertzian growth kinetics and all types of cancerous cells have a common carrying capacity. On the contrary, the model assumes that non-cancerous precursor cells are growing logistically, which can be mathematically represented as $\propto_{N}*N\left(1-\rho \right)*\left(1-\frac{N}{K}\right)$ (36, 37).

#### Model Equations

Based on the biological significance and mathematical assumptions, four ODEs were developed for four different types of tumor cells using 29 parameters (Supplementary Table 2) in order to describe evolutionary dynamics of tumor growth. The model equations have been listed below:

(1) $$\begin{array}{l} \frac{dN}{dt}=\propto_{N}*N\left(1-\rho \right)*\left(1-\frac{N}{K}\right)-\delta_{N}N-{\;\propto }_{N}*N*\rho \end{array}$$

(2) $$\begin{array}{l} \frac{dC}{dt}=\;\propto_{N}*\rho *N+\alpha_{C}*C*\text{log }\frac{K}{C+\mu }\\ \;-\gamma_{R}\alpha_{C}C-\omega_{S}\alpha_{C}C\;-\;\delta_{c}C \end{array}$$

(3) $$\begin{array}{l} \frac{dC_{R}}{dt}=\gamma_{R}\alpha_{C}C+\bar{\gamma_{R}}\alpha_{CS}C_{s}+\alpha_{CR}*C_{R}*\text{log }\frac{K}{C_{R}+\mu }\\ \;-\delta_{R}C_{R}-\bar{\omega_{S}}\alpha_{CR}C_{R} \end{array}$$

(4) $$\begin{array}{l} \frac{dC_{s}}{dt}=\bar{\omega_{S}}\alpha_{CR}C_{R}+\omega_{S}\alpha_{C}C+\alpha_{CS}*C_{s}*\text{log }\frac{K}{C_{S}+\mu }\\ \;-\delta_{S}C_{S}-\bar{\gamma_{R}}\alpha_{CS}C_{s} \end{array}$$

#### Parameter Estimation and Validation of Tumor Growth Model

For the simulation and validation of the tumor growth dynamics, the calibration of the model has been carried out and the unknown parameters present in the model have been estimated by appropriately fitting the initial growth kinetics of the model with experimental data of drug sensitive and resistant cell lines of Glioma origin (without the effect of any drugs) for 96 h (38–40). The parameter estimation was performed using the MATLAB based toolbox that uses Markov Chain Monte Carlo (MCMC)-DRAM algorithm (41). Trace plots and Histograms are given in Supplementary Figure 1. For this purpose, the U-87 cell growth data has been considered as drug sensitive Glioma cells as U87 cells express low level of MGMT (42). On the other hand, T-98 has been considered as drug resistant cell line due to high expression of cystine-glutamate exchanger (xCT), MGMT, NAMPT (nicotinamide phosphoribosyl transferase), BER (base excision repair), ubiquitin-specific peptidase (Usp18) etc. (42). These protein expressions have been correlated in literature with activity of the transporters (influx, efflux), metabolism, increased DNA repair activity and other processes leading to drug resistance (43).

In this study, the tumor growth model (without drug) consists of four variables and 15 parameters. Out of these values, three parameter values are known from experiments and literature, 10 parameters were estimated using the MCMC technique, and other two parameters were assumed and labeled as expected values as these have been estimated to obtain a close fit of all the four variables during the model simulation The MATLAB codes for performing the parameter estimation have been made available in Supplementary Text 2 (Scripts 1–4). The model contains additional 14 parameters related to TMZ and the Acridone derivative that regulates drug response behavior of the model (Section model initialization and numerical simulations). The parameter values used for the simulation have been enlisted in Supplementary Table 2.

#### Model Initialization and Numerical Simulations

Using the parameter values and initial values, the system of ODEs were solved numerically utilizing Runge-Kutta methods (RK). In this model, two types of cells non-cancerous cells (N) and Cancer cells (C) have been considered. The C is thought to be consisting of subpopulations-Drug resistant cancer cells (C_R_) and Drug sensitive cancer cells (C_S_). Numerical simulation of this model was carried out using ODE45, variable order solver present in MATLAB® 2017a platform. The model was initialized and simulated by considering initial values of N, C, C_R_, and C_S_ similar to that of the initial values considered in experiments (Supplementary Table 3).

#### Incorporation of Effects of TMZ and Acridone Derivative

After validating the natural growth dynamics of the tumor, without incorporating the effect of any drug, the model equations have been modified with additional terms to capture the cell kill dynamics under the influence of individual drugs (TMZ and Acridone derivative) and when administered in combination. For this, the Maximum response (E_max_) model for drug induced cell death has been considered in our model for comparing efficacy of the drugs. Functional form of E_max_ model along with an additional parameter (Hill exponent to show steeper relationship of concentration) to the concentration can be depicted as $\left[\frac{\varepsilon_{\max}{\left(Conc\right)}^{Hill}}{{C_{50}}^{Hill}+\;Conc^{Hill}}\right]$, where *C*_50_ is the concentration at 50% of E_max_ (44, 45)_._ Similarly, effects of drugs on two different cancerous cells have been introduced in this mathematical model. In order to capture this, **Equations 3, 4** have been modified as follows:

(5) $$\begin{array}{l} \frac{dC_{R}}{dt}=\gamma_{R}\alpha_{C}C+\bar{\gamma_{R}}\alpha_{CS}C_{s}+\alpha_{CR}*C_{R}*\text{log }\frac{K}{C_{R}+\mu }\\ \;-\delta_{R}C_{R}-\bar{\omega_{S}}{\;\alpha }_{CR}C_{R}-\left[\varepsilon_{\max}^{D1r}\frac{D_{1}^{\eta_{D1r}}}{IC_{50D1r}^{\eta_{D1r}}+D_{1}^{\eta_{D1r}}}\right]C_{R}\\ \;-\left[\varepsilon_{\max}^{D2r}\frac{D_{2}^{\eta_{D2r}}}{IC_{50D2r}^{\eta_{D2r}}+D_{2}^{\eta_{D2r}}}\right]C_{R} \end{array}$$

(6) $$\begin{array}{l} \frac{dC_{s}}{dt}=\bar{\omega_{S}}\alpha_{CR}C_{R}+\omega_{S}\alpha_{C}C+\alpha_{CS}*C_{s}*\text{log }\frac{K}{C_{S}+\mu }\\ \;-\delta_{S}C_{S}-\bar{\gamma_{R}}\alpha_{CS}C_{s}-\left[\varepsilon_{\max}^{D1s}\frac{D_{1}^{\eta_{D1s}}}{IC_{50D1s}^{\eta_{D1s}}+D_{1}^{\eta_{D1s}}}\right]C_{S}\\ \;-\left[\varepsilon_{\max}^{D2s}\frac{D_{2}^{\eta_{D2s}}}{IC_{50D2s}^{\eta_{D2s}}+D_{2}^{\eta_{D2s}}}\right]C_{S} \end{array}$$

Here D_1_ is the dose of the TMZ in μ*M*, while D_2_ is the dose updated every time with the parameter values of the Acridone derivatives for AC2, AC7, and AC26 in μ*M*, ε_max_ is given for Drug 1 and Drug 2 in different cancer cells (Drug resistant cancer cells and drug sensitive cancer cells), η_*D*1*r*_ and η_*D***2***r*_ (Hill exponents) represents efficacy of the Drug 1 and Drug 2 in for resistant cancer cells (similarly η_*D*1*s*_ and η_*D*2*s*_ are for sensitive cells), while IC_50_ stands for inhibitory concentration of Drug 1 and Drug 2 at which 50% of tumor response is inhibited. After incorporation of the effect of drugs (TMZ and Acridone derivative) the model now consists of 29 parameters. However, it is to be noted that each of the parameters related to the Acridone derivative (Drug 2 or D_2_) can have three possible values related to AC2, AC7, and AC26, thereby making the total number of possible values of the parameters 43 (Supplementary Table 2, Supplementary Text 2: Scripts 5 and 6). Here, the doses for the four drugs (TMZ, AC2, AC7, and AC26) were varied to study the change in cellular dynamics under different dosages. The parameter values related to these drugs considered in our model equations have been determined by varying the parameters to obtain a close fit with the experimental observations from the SRB Assay.

#### Numerical Simulation With Drug Therapy

Numerical simulations were carried out by varying the dose of the drug (D_1_) [i.e., TMZ while keeping the dose of the Acridone derivative drug (D_2_) as zero]. Simulations were run using an ODE variable solver in MATLAB until a steady state solution was reached. For the study of the effect of the individual drugs on the inhibition of cellular growth, simulations for AC2, AC7, and AC26 were also performed in a similar way by varying the dose of the Acridone derivatives. These simulation results in capturing the effect of the individual drugs on the drug sensitive and resistant cell lines were fitted and validated with our experimental findings obtained from the Sulforhodamine B Assay mentioned in Section Sulforhodamine B assay for individual drugs.

#### Administration of Combinatorial Drugs

After studying the effect of drugs individually on the growth of different Glioma cells, combinational studies were carried out to observe how the growth inhibition of TMZ resistant Glioma cells could be achieved when TMZ and Acridone derivatives were administered in combination. For this, combinatorial drugs (AC26 + TMZ, AC2 + TMZ, and AC7 + TMZ) were administered and their effect on growth of drug resistant cancer cells and drug sensitive cancer cells were observed. Simulations were performed by varying doses of both the drugs for each combinational drug within the range that would not lead to drug toxicity. Toxicity range was considered as more than 100 μ*M* for Acridone compounds and more than 250 μ*M* for TMZ (42). Also, because it was observed from the experiment and the previous simulations that Acridone compounds were highly cytotoxic and potent at lower concentrations so the range of Acridone moieties in simulations were varied from 0 to 6 μ*M* only. For each of the drugs, both TMZ and the Acridone derivatives, 100 dosages were considered and simulated using our mathematical model to generate 100 × 100 = 10,000 dosage combinations *in silico* (Supplementary Text 2: Scripts 7 and 8). The dose response matrix of C_R_ and C_S_ generated from the simulation was used to calculate the Synergy Scores (SC) of each combination.

#### Sensitivity Analysis

In order to determine the effect of the different model parameters and the dosages of TMZ and Acridone derivatives on the model variables and the dose response matrices, the Sensitivity Analysis was performed using LHS-PRCC (Latin Hypercube Sampling - Partial Rank Correlation Coefficient) method in MATLAB (46). This method is useful for global uncertainty analysis for monotonic systems. The sensitivity analysis was performed on 29 parameters including the parameters related to TMZ (D1) and Acridone derivatives (D2). The parameters range related to the Acridone derivatives (D2) were considered such that it covers the values of all the three derivatives, viz., AC2, AC7, and AC26. The sensitive parameters were identified for all the four variables based on their PRCC values with a *p* < 0.05 (Supplementary Text 2: Scripts 10–12).

#### *In-silico* Drug Synergy Estimation

The synergy scores (SC) for each pair of drug combinations in the resistant cells were calculated using the R-based package Synergy Finder using the Bliss reference model (47). The dose-response matrix obtained from the numerical simulation of the model for the C_R_ cells was used as an input matrix (100 × 100) for Synergy Finder to calculate the synergy scores (SC). For the calculation of synergy scores, the assumption is that if an experiment drug A at dose x1 is combined with drug B at dose x2, then the effect of such a combination is y_c_ as compared to the monotherapy effect of A at x1 and B at x2. In order to quantify the degree of synergy, the value of y_c_ needs to be compared with the expected effect y_e_ of non-interaction. In the Bliss model y_c_ is calculated as

(7) $$\begin{array}{l} y_{\text{C}}=y_{\text{A}}+y_{\text{B}}-y_{\text{A}}y_{\text{B}} \end{array}$$

The synergy score is calculated as the difference between the observed effect (y_C_) and the expected effect (y_e_). This method has been used when the two drugs are acting independently on the phenotype.

### Sulforhodamine B Assay for Combinatorial Drugs

Sulforhodamine B Assay procedure used for evaluation of combinatorial drugs is the same as that of the individual drugs. Cell lines were treated with various concentrations of drug combinations TMZ+AC2, TMZ+AC7, and TMZ+AC26 instead of individual drugs. Combination index (CI) of these combinatorial drugs were evaluated in drug-sensitive (U-87) and drug-resistant (T-98) cancer cell lines. The 100 μ*M* concentration of TMZ with IC_10_ concentration of the AC2, AC7, and AC26 were used for the combination assay. Based on CI values obtained for these drug combinations, it is possible to determine the type of interaction. If CI value is <0.8, then combinatorial drugs show synergism (i.e., its effect is better than the expected theoretical effect); when CI value lies between 0.8 and 1.2, combinatorial drugs show additive behavior i.e., it means the effect of combination will be equal to sum or product of each separate effect; and when CI value is more than 1.2, combinatorial drugs show antagonism i.e., its effect is worse than expected theoretical effect (48, 49).

### Acute Toxicity Study of Acridone Derivatives

Animal study was conducted for acridone derivatives AC2, AC7, and AC26 alone and in combination with Temozolomide for Acute Oral Toxicity - Fixed Dose procedure. The Institutional Animal Ethics Committee bearing CPCSEA/IAEC/P-52/2016 was approved for undertaking the study. Acute oral toxicity was conducted on female albino Wistar rats for 8–12 weeks and were maintained at 25 ± 2^0^C in a conditioned room with 50–60% of humidity and free access to food and water was given. Rats were kept for fasting overnight (12 h) before dosing and weights were recorded periodically. Rats in two groups were given acridone derivatives alone at a dose of 300 and 2,000 mg, rats in two groups were given a combination of Temozolomide : acridone derivatives at dose of 10:1 mg/kg and 15:1.5 mg/kg body weight and one group was kept as control. Dose of Temozolomide was taken as per previously published article (50). Compounds alone and combination were suspended in 2.0% of Tween 80 in normal saline and control group was taken for vehicle only. After administering the compound, food was not provided for 3 h. All the rats were monitored periodically for 1 h upto 12 h on the 1st day and thereafter twice in a day for mortality, behavioral changes, signs and symptoms of toxicity for 14 days. Individual weights of rats were taken for all the 14 days and study was conducted twice for each dose.

## Results

### Characterization of the Drugs

Successful delivery of any anti-cancer drug across the blood brain barrier depends mainly on the lipophilicity of the drug (51). The Acridone derivatives (AC2, AC7, and AC26) are weak bases existing in both the uncharged (unprotonated) and charged (protonated) forms (52). Characteristics of novel Acridone derivatives like molecular weight, melting point, Log P (or partition co-efficient) and pKa have been evaluated and results of the same are shown in Table 1. It seems that the lipophilicity of the compounds contributes to the anti-neoplastic activity to some extent. The results from the Online BBB Predictor show that all the three Acridone derivatives AC2, AC7, and AC26 have blood brain barrier permeability with BBB scores 0.238, 0.236, and 0.144, respectively. On the other hand, the tool predicts TMZ to have a BBB score of 0.034 (Supplementary Figure 3) (17).

**Table 1:** Physical data for acridone derivatives.

**Compound name**	**Molecular formula**	**Molecular weight (g/mol)**	**Melting point (^**0**^C)**	**Log *P***	**pKa**
AC-26	C_22_H_17_N_3_O_3_	371.39	246–250	3.17	6.517
AC-2	C_23_ H_17_N_3_O_2_	367.40	238–242	3.92	8.028
AC-7	C_21_H_14_ClN_3_O_2_	375.81	129–134	4.54	9.234

### Molecular Docking of Acridones With P-gp, MRP, and MGMT

In order to determine the binding affinities of these Acridone derivatives with drug resistant causing proteins P-gp, MRP, and MGMT, Molecular docking studies have been performed. Acridone derivatives are very much recognized as substrates of efflux pumps P-gp and MRP proteins with potential DNA intercalating property for multidrug resistant (MDR) cancers (52). Acridones being substrates or inhibitors of these efflux pumps enhances the concentration of drugs like Temozolomide inside the cell, which can lead to cell death. Also the combination of anticancer drugs with acridone derivatives can modulate or prevent the cause of drug resistance (53). Same hypothesis might have improved the cytotoxicity against drug resistant cancer cells in SRB assay. To further verify the experimental results and predict the binding affinity of acridone derivatives, molecular docking was performed against P-gp, MRP, and MGMT target proteins.

**Figure 3:**
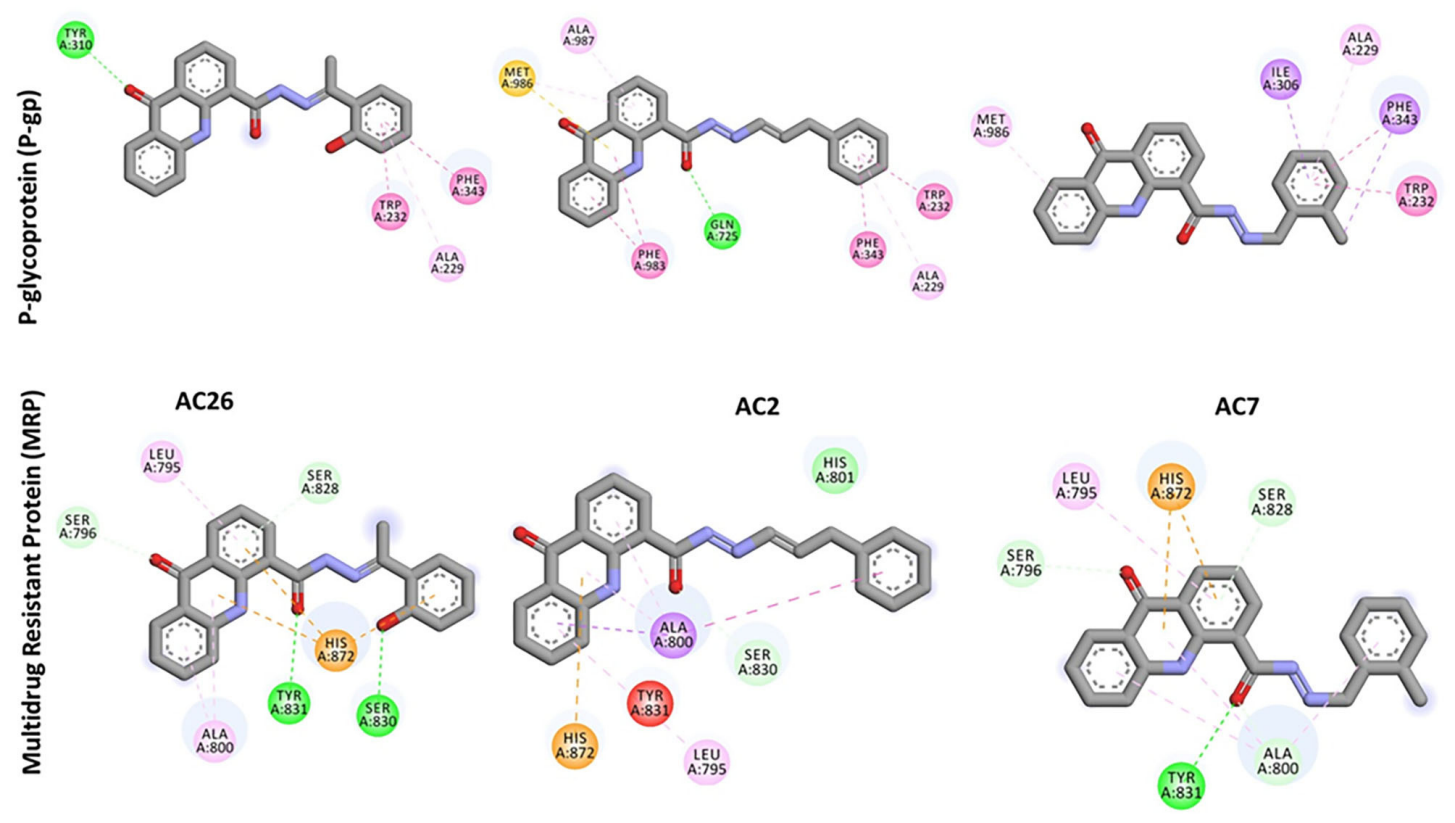
2D docked poses of AC26, AC2, and AC7 with P-gp and MRP. The interactions shown in the figure have been color coded as Green: Hydrogen bonding; Yellow: Pi-Sulfur; Pink: Pi-Pi Stacking; Light Pink: Pi-Alkyl; Blue: Pi-Sigma.

Docking of Acridones with P-gp has identified AC26 with highest binding affinity of −10.2 kcal/mol (Table 2). Complex was found stabilized by hydrogen bonding with TYR A:310; Pi-Pi Stacking with TYR A:232, PHE A:343, and Pi-Alkyl interactions with ALA A:229 residues (Figure 3). The AC2 has demonstrated binding affinity of −10.1 kcal/mol by hydrogen bonding with GLN A:725, Pi-Pi Stacking with TRP A:232; PHE A:343; PHE A:983, Pi-Sulfur with MET A:986, and Pi-Alkyl interactions with ALA A:229, ALA A:987 residues. Most of the interactions were found common with AC26 unlike hydrogen bonding and Pi-Sulfur interacting residues. Surprisingly, AC7 was found stabilized with −9.5 kcal/mol without hydrogen bonding interactions. All the three compounds have interacted with different binding patterns and interactions with P-gp. Complex structure and size of active pocket might have led to different interactions.

**Table 2:** Docking of acridones with P-gp and MRP.

**S. No**.	**Target**	**Compound code**	**Binding affinity (kcal/mol)**	**Interactions at the active pocket**	
				**Type of interactions**	**Residue information**
1	P-glycoprotein (P-gp)	AC26	−10.2	Hydrogen bonding Pi-Pi Stacking Pi-Alkyl	TYR A:310 TRP A:232; PHE A:343 ALA A:229
2		AC2	−10.1	Hydrogen bonding Pi-Pi Stacking Pi-Sulfur Pi-Alkyl	GLN A:725 TRP A:232; PHE A:343; PHE A:983 MET A:986 ALA A:229; ALA A:987
3		AC7	−9.5	Pi-Sigma Pi-Pi Stacking Pi-Alkyl	ILE A:306; PHE A:343 TRP A:232 ALA A:229; MET A:986
1	Multidrug Resistance Protein (MRP)	AC26	−7.5	Hydrogen bonding Pi-Anion Pi-Alkyl	SER A:796; SER A:828; SER A:830; TYR A:831 HIS A:872 LEU A:795; ALA A:800
2		AC2	−7.1	Van Der Waals Hydrogen bonding Pi-Pi Stacking Pi-Anion Pi-Sigma Pi-Alkyl	HIS A:801 SER A:830 ALA A:800 HIS A:872 ALA A:800 LEU A:795
3		AC7	−7.2	Hydrogen bonding Pi-Anion Pi-Alkyl	SER A:796; ALA A:800; SER A:828; TYR A:831 HIS A:872 LEU A:795

Similarly, docking of Acridones with MRP has identified AC26 with binding affinity of −7.5 kcal/mol (Table 2). Interestingly, complex was found stabilized by hydrogen bonding with four residues [i.e., SER A:796; SER A:828; SER A:830; TYR A:831, Pi-anion with HIS A:872, and Pi-alkyl interactions with LEU A:795; ALA A:800 (Figure 3)]. AC2 and AC7 have exhibited −7.1 and −7.2 kcal/mol. Only AC2 has formed Van der Waals interaction with HIS A:801. Pi-alkyl interaction with LEU A:795 and Pi-anion with HIS A:872 was found common with all the three compounds. AC7 also demonstrated four hydrogen bonding interactions. Unlike AC26 and AC2 hydrogen bonding with SER A:830 was found missing with AC7. This particular interaction might have reduced the binding affinity to −7.2 kcal/mol. All the three compounds have exhibited a good number of interactions at the active pocket of MRP.

**Figure 4:**
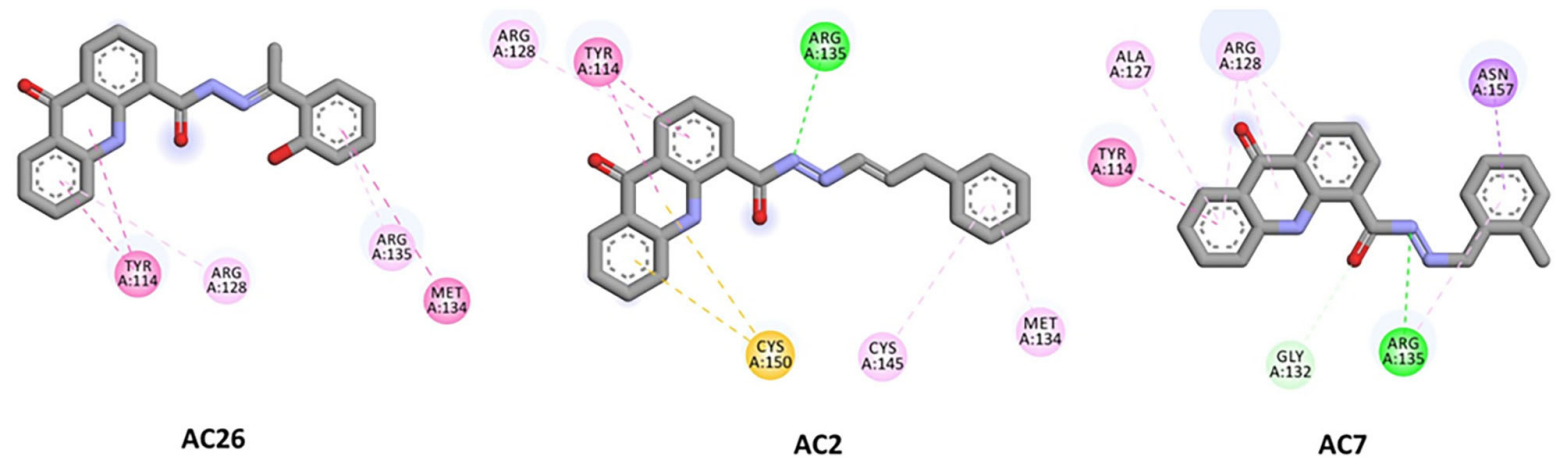
2D docked poses of AC26, AC2, and AC7 with MGMT. The interactions shown in the figure have been color coded as- Green: Hydrogen bonding; Yellow: Pi-Sulfur; Pink: Pi-Pi Stacking; Light Pink: Pi-Alkyl; Blue: Pi-Sigma.

Finally, docking of Acridones with MGMT has revealed interesting insights. The AC2 and AC26 were found stabilized with good binding affinity of −7.8 and −7.7 kcal/mol (Table 3). Also AC2 has demonstrated hydrogen bonding with ARG A:135, Pi-Pi stacking with TYR A:114, Pi-alkyl with ARG A:128; MET A:134; CYS A:145, and Pi-sulfur with CYS A:150. Only AC2 has formed Pi-sulfur interaction among the three compounds (Figure 4). AC7 has also exhibited hydrogen bonding interactions with ARG A:135; GLY A:132, and other Pi-Pi stacking, Pi-alkyl, Pi-sigma interactions at the active pocket of MGMT. Also, only compounds formed Pi-sigma interactions. Surprisingly, AC26 was found stabilized by Pi-Pi Stacking with TYR A:114; MET A:134 and Pi-alkyl with ARG A:128; ARG A:135 interactions. Compound has shown −7.7 kcal/mol binding affinity with no hydrogen bonding interactions. Pi-Pi stacking with TYR A:114 and Pi-alkyl with ARG A:128 were found common with all the three compounds.

**Table 3:** Docking of acridones with MGMT.

**S. No**.	**Compound code**	**Binding affinity (kcal/mol)**	**Interactions at the active pocket**	
			**Type of interactions**	**Residue information**
1	AC26	−7.7	Pi-Pi Stacking Pi-Alkyl	TYR A:114; MET A:134 ARG A:128; ARG A:135
2	AC2	−7.8	Hydrogen bonding Pi-Pi Stacking Pi-Alkyl Pi-Sulfur	ARG A:135 TYR A:114 ARG A:128; MET A:134; CYS A:145 CYS A:150
3	AC7	−7.1	Hydrogen bonding Pi-Pi Stacking Pi-Alkyl Pi-Sigma	ARG A:135; GLY A:132 TYR A:114 ALA A:127; ARG A:128 ASN A:157

Overall, docking calculations revealed that acridones have good binding affinity to P-gp and MGMT. Compounds have exhibited lesser binding affinity with MRP compared to other targets. Particularly, AC26 has demonstrated highest binding affinity with all the three target proteins. Molecular docking studies of acridones against P-gp target has revealed that compounds have the ability to modulate or reverse drug resistance mediated by efflux pumps like P-gp with good binding affinity. Docking studies have once again supported that Acridones are known to modulate MDR as P-gp substrate/inhibitor. Study suggests that Acridone derivatives can be further optimized for the design of safe and potent MGMT inhibitors.

### Sulforhodamine B Assay for Individual Drugs

The cell growth inhibition potential of the Acridone derivatives on the drug sensitive and drug resistant Glioma is studied using the Sulforhodamine B (SRB) assay. Here the IC_50_ (μM) values for the drugs AC2, AC7, AC26, and TMZ were determined and tabulated in Table 4. We have found out IC_50_ values of acridone derivatives using SRB Assay and have confirmed safety dose by conducting acute toxicity studies on albino Wistar rats. However, the reported IC_50_ values of acridone derivatives are based on *in vitro* study only and is yet to be tested for clinical application. Also, this experimental data was used to plot growth inhibition curves for AC2, AC7, AC26, and TMZ in U-87 drug sensitive Glioma cell lines and T-98 TMZ resistant Glioma cell lines (Figure 5). All the four compounds AC26, AC2, AC7, and TMZ were found active. Here we observe that the compound AC26 containing substituent like 1-(2-hydroxyphenyl) ethylidene have shown better results in both drug resistant and sensitive types of Glioma cell lines (U-87 and T-98). Results show that this substitution is responsible for high anti-proliferative activity. Also, substitution of –Cl in phenyl group in AC7 was found to overcome drug resistance to a larger extent in comparison to having no substitution in phenyl group in AC2. One important aspect related to AC7 is that the presence of –Cl group in the phenyl group is also one of the main reasons for the drug not being effective on U-87. From this we observe that in comparison to other Acridone derivatives, AC26 showed highest cytotoxicity when compared with AC7 and AC2, respectively.

**Table 4:** Dose-effect relationships of TMZ and AC in Glioma cell lines^#^.

**Name of cell lines**	**Vero cell Line**	**U-87/WT**		**T-98/TMZRES**	
**Drugs**	**CC_**50**_(μM)**	**IC_**50**_ (μM)**	**SI**	**IC_**50**_ (μM)**	**SI**
TMZ	482	23.33 ± 7.57	20.66	190 ± 0.16	2.53
AC26	66.08	0.9 ± 0.1	73.42	0.76 ± 0.053	86.94
AC2	56.21	1.53 ± 0.85	36.73	1.53 ± 0.25	36.73
AC7	48.17	5.67 ± 0.58	8.49	1.05 ± 0.18	45.87

# *Selectivity Index (SI) = CC_50_ of Vero cell line /IC_50_ of Cancer cell line after 72 h of TMZ or AC*.

#*IC_50_ values are represented in the form of Mean ± Standard Deviations for both the cell lines*.

**Figure 5:**
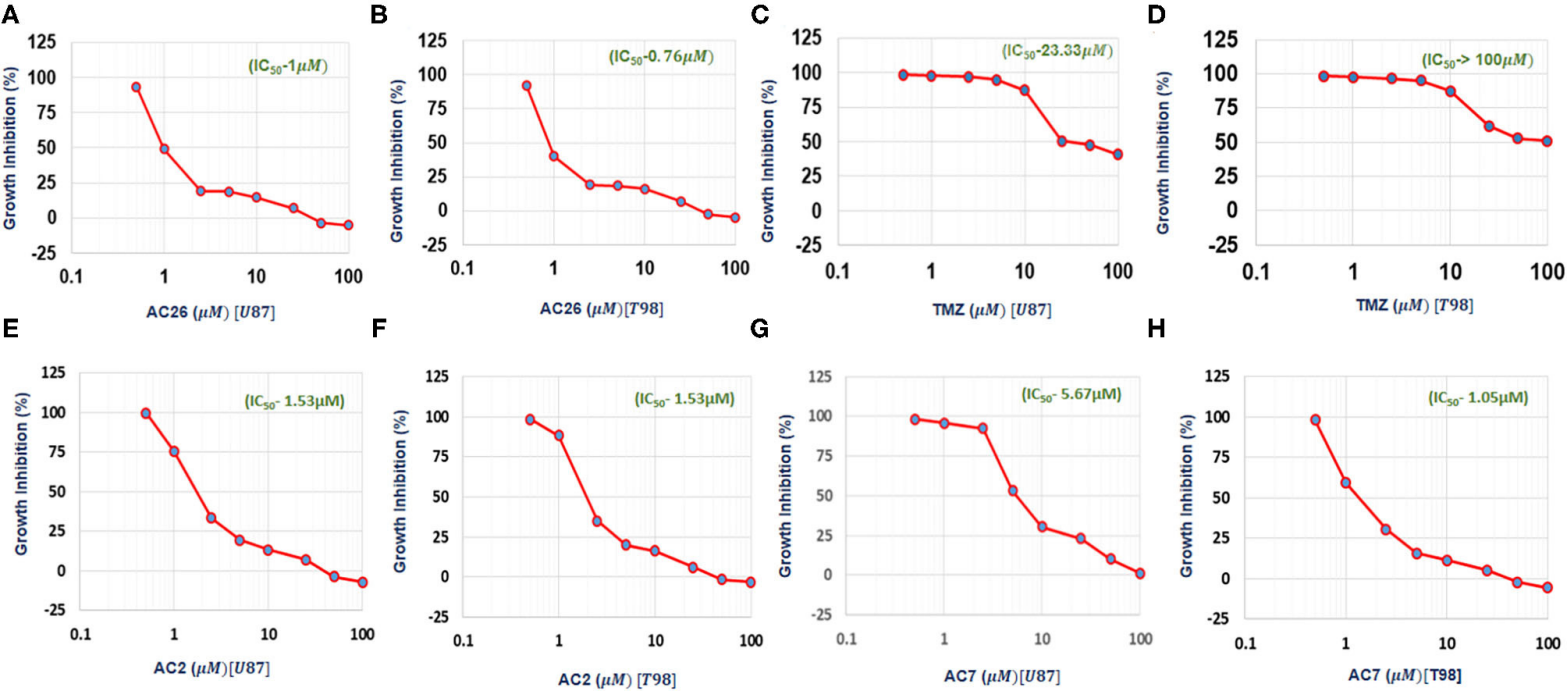
IC_50_ values obtained from experimental data: **(A)** Growth Inhibition Curve for AC26 in U-87 Glioma Cell Lines; **(B)** Growth Inhibition Curve for AC26 in T-98 Glioma Cell Lines; **(C)** Growth Inhibition Curve for TMZ in U-87 Glioma Cell Lines; **(D)** Growth Inhibition Curve for TMZ in T-98 Glioma Cell Lines; **(E)** Growth Inhibition Curve for AC2 in U-87 Glioma Cell Lines; **(F)** Growth Inhibition Curve for AC2 in T-98 Glioma Cell Lines; **(G)** Growth Inhibition Curve for AC7 in U-87 Glioma Cell Lines; **(H)** Growth Inhibition Curve for AC7 in T-98 Glioma Cell Lines. X axis showing log concentration of the drug in μM.

### Selectivity Index

Selectivity indices (SI) of all the four drugs, were obtained from the experiments, have been tabulated (Table 4). A drug with high SI is able to act against cancer cells effectively at concentrations below its cytotoxic concentration. The SI data shows that AC26 is more selective toward T-98 (drug resistant cancer cell line) and U-87 (drug sensitive cancer cell lines) than its selectivity toward Vero cell lines. These results indicate the supremacy of AC26 as a better choice of drug for treatment of cancer that would be effective both on T-98 and U-87 drug lines.

### Numerical Simulations and Parameter Estimation (Without Drug)

**Figure 6:**
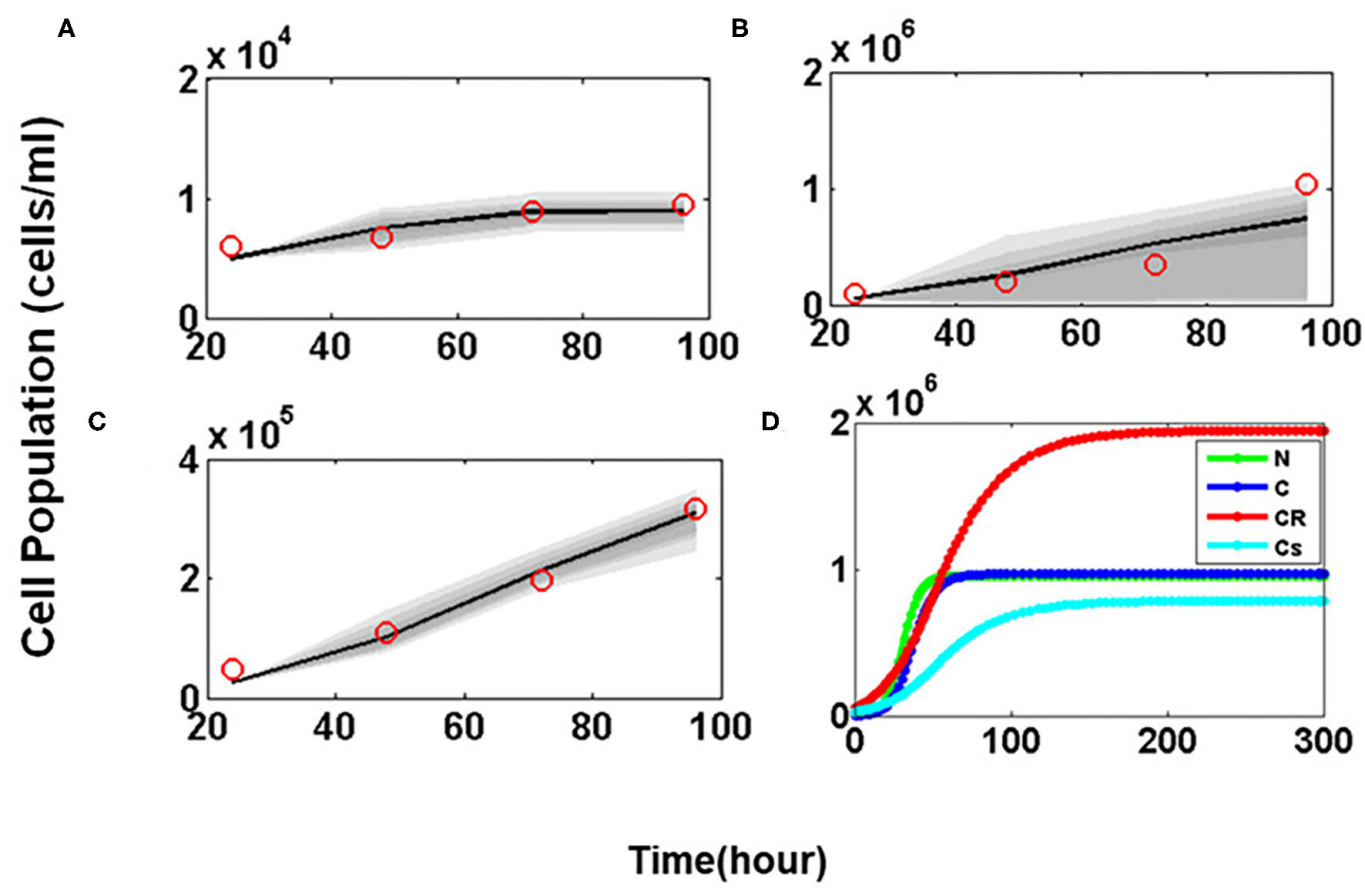
Parameter estimation and validation studies. Predictive Plots using estimated parameter values for **(A)** Cancer cells (C), **(B)** Drug resistant cancer cells (C_R_) and **(C)** Drug sensitive cancer cells (C_S_). The red circle in predictive plots represents cell proliferation values obtained from Glioma cell line data; **(D)** Temporal Dynamics shown by Normal (N), Cancer cells (C), Drug resistant cancer cells (C_R_), and Drug sensitive cancer cells (C_S_).

In order to mimic the experimental observations *in silico*, first the Glioma cell growth model was developed without the administration of any drug (**Equations 1–4**). The unknown values of 10 parameters were estimated by MCMC method. Sensitive parameters regulating the growth of the variables C, C_R_, and C_S_ were varied within biologically feasible ranges. Time course cell growth experimental data of 96 h for Glioma, U-87 and T-98 cells lines were used to fit the model parameters (red circles, Figure 6) (29–31). It was assumed that the prior distribution is normal. The MCMC simulation was run for 500,000 iterations to assure convergence of the chain. The final parameter values estimated by MCMC algorithm for the mathematical model have been listed in Supplementary Table 2. The simulated predictive plots, with the estimated parameters, obtained for cancer cells C (Figure 6A), Cancer Resistant C_R_ (Figure 6B), and Cancer Sensitive cells C_S_ (Figure 6C) show a good fit with the experimental data points (indicated with red circles) and mostly lie within the 95% confidence interval. This ensures the validity of the parameter chosen and the mathematical model for closely mimicking the *in vitro* experiments.

The model was then simulated for 300 h to ensure that all four variables representing the tumor sub-populations reach steady state. Figure 6D shows the temporal dynamics of all four of cellular sub-populations. Here it is observed that at steady state the C_R_ population reaches a much higher concentration as compared to the C_S_ cells which makes the tumor resistant to therapeutic interventions.

### Numerical Simulations With TMZ and the Combinations

After successful validation of the tumor cell growth model, it is now used to test the cellular inhibition effect of the drugs individually and then in combinations. Experimental data from the SRB assay was used to calibrate and validate the model outcome.

#### Administration of Individual Drugs

**Figure 7:**
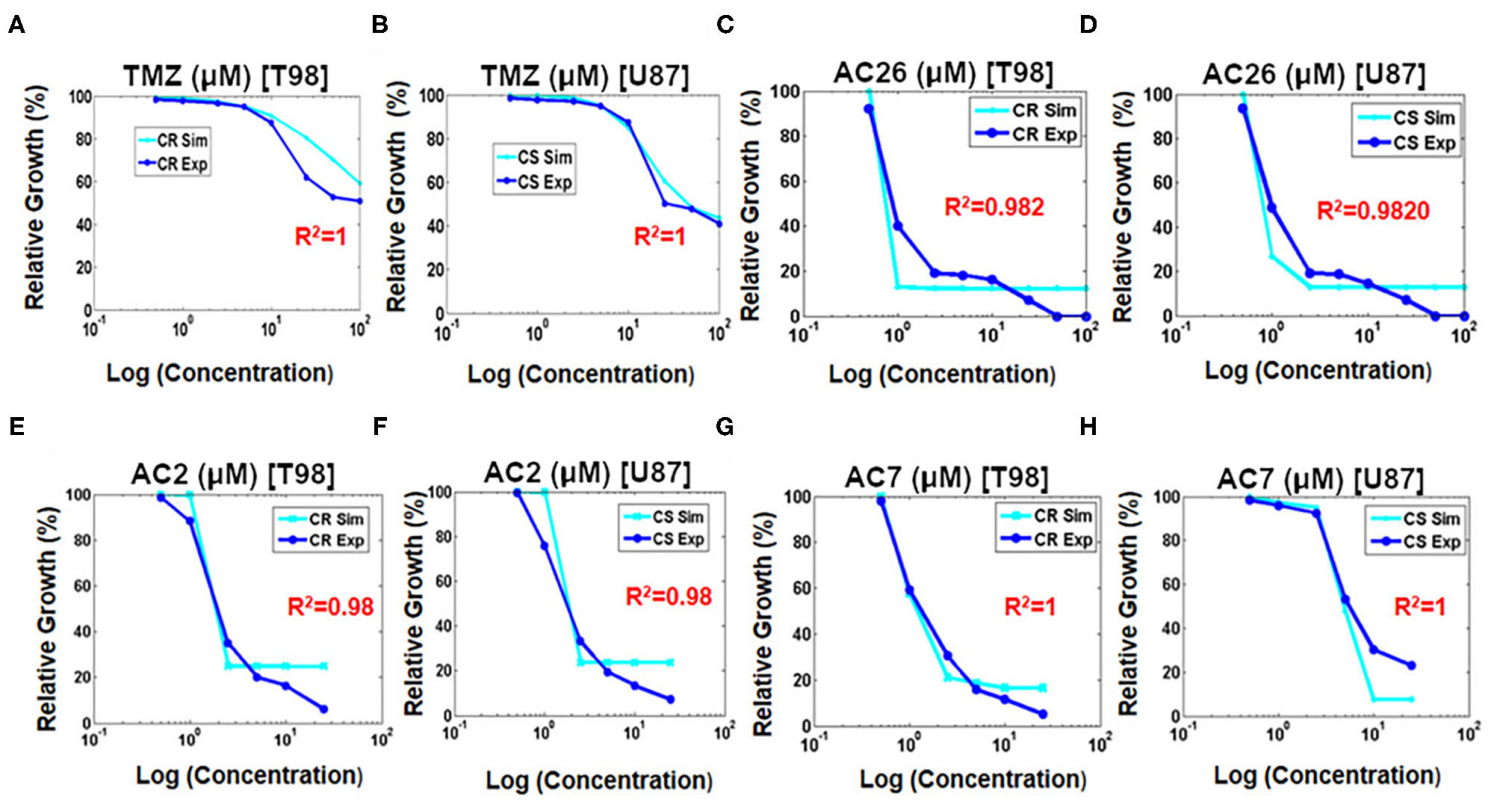
Fitting growth inhibition curves of experimental data with simulated data after administration of drugs on cancer resistant C_R_ (T-98) and sensitive cell lines C_S_ (U-87). **(A)** TMZ to C_R_ (T-98); **(B)** TMZ to C_S_ (U-87); **(C)** AC26 to C_R_ (T-98); **(D)** AC26 to C_S_ (U-87); **(E)** AC2 to C_R_ (T-98); **(F)** AC2 to C_S_ (U-87); **(G)** AC7 to C_R_ (T-98); **(H)** AC7 to C_S_ (U-87).

Numerical simulations were performed to study the growth inhibition of the drug sensitive C_S_ and drug resistant C_R_ cancer cells by varying the doses of TMZ, AC26, AC2, and AC7 individually. The Figures 7A,B obtained shows relative tumor growth (%) with the changing concentration of TMZ. Here, it can be observed that both the simulated data (cyan) and experimental data (royal blue) closely fit. The spearman's rank-order correlation *R*^2^ values for both drug resistant cancer cells (T-98) and drug sensitive cancer cells (U-87) indicating very strong correlation between both the experimental data and simulated results. The IC_50_ values obtained from the simulations also match with the experimental data pretty well.

Similarly, the growth inhibition effect of the Acridone derivatives AC26 (Figures 7C,D), AC2 (Figures 7E,F), and AC7 (Figures 7G,H) were also studied on the drug resistant C_R_ and drug sensitive C_S_ cancer cells. The simulation results show a good fit of experimental data for all the three drugs (*R*^2^ > 0.98).

#### Administration of Combinatorial Drugs—TMZ With Acridone Derivative

**Figure 8:**
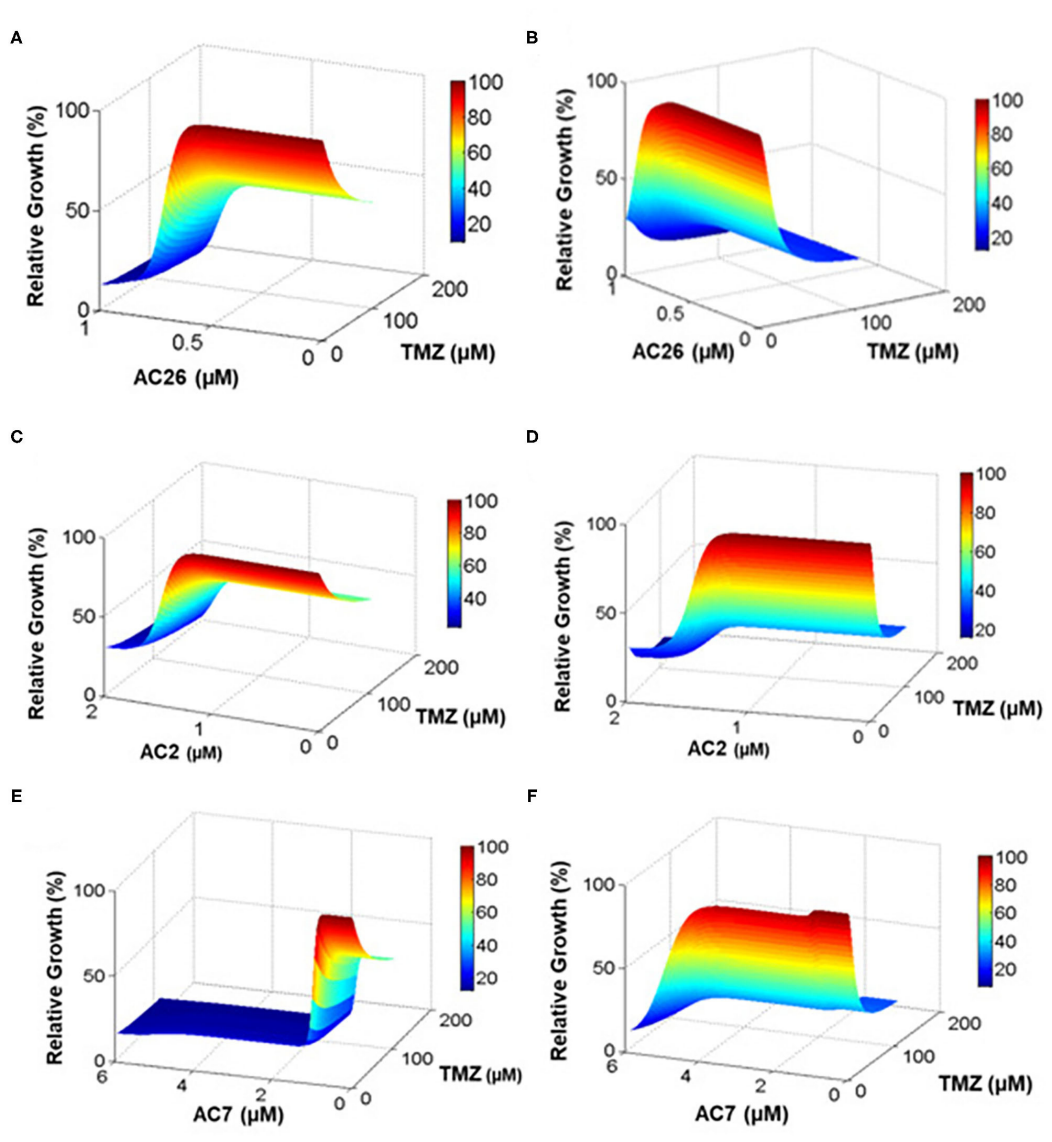
Drug dosage variation study. The percentage relative growth of drug resistant (CR) and sensitive cancer (CS) cells growth under varying drug combinations: **(A)** Resistant cells with TMZ and AC26; **(B)** Sensitive cells with TMZ and AC26; **(C)** Resistant cells TMZ and AC2; **(D)** Sensitive cells with TMZ and AC2; **(E)** Resistant cells with TMZ and AC7; **(F)** Sensitive cells with TMZ and AC7. The figure represents the relative growth (%) for 10,000 dosage combinations for each pair of drugs.

Various doses of combinatorial drugs D_1_ -TMZ and D_2_- Acridone derivatives (AC26/AC2/AC7) were administered to the model and the simulations were run until steady state was reached. The dose response matrix showing the relative growth percentage (along Z-axis) of drug resistant cancer cells and drug sensitive cancer cells have been depicted through surface plots (Figure 8) for 10,000 dosage combinations of each pair of TMZ and Acridone derivative (AC26/AC2/AC7). The Figure 8A shows that when dose of D_1_ (TMZ) is 0, tumor growth is maximum (represented by red color), but as dose is increased slowly from 0 to 200, the TMZ inhibits tumor growth reduction by 50%. Here we also observe that, alongside TMZ, as dose of D_2_ (i.e., AC26) is increased, drastic fall in the number of drug resistant cancer cells is observed (more than 20% tumor growth reduction). While comparing the efficacy of both the drugs, it was observed that AC26 was more successful in inhibiting drug resistant cancer cell growth at lower concentration than TMZ. Conversely, Figure 8B shows that increase in the dose of TMZ results in sudden fall in concentration of drug sensitive cancer cells (40% tumor growth reduction) whereas increase in the dose of AC26 leads to moderate reduction in drug sensitive cancer population (10–20% of tumor growth reduction).

Similarly, the dosage of the drugs TMZ and AC2 were varied from 0 to 200 and 0 to 2, respectively (Figures 8C,D), while in the third drug combination study, TMZ and AC7 were varied from 0 to 200 and 0 to 6, respectively (Figures 8E,F). In both the cases we observed a better growth inhibition of the resistant tumor cells on administration of the Acridone derivative AC2 and AC7 when combined with TMZ as opposed to when they were administered individually. From the dose response matrices, the fold change in the IC_50_ values of TMZ and the Acridone derivatives when used in combinations as opposed to when they are administered individually were also calculated (**Table 6**). Here we observe that when the drugs are used in combination, the IC_50_ value of TMZ and Acridone derivatives reduces substantially. This observation throws light on the possibility of existence of synergistic effects of the drugs when used in combination with TMZ in the drug resistant cancer cells.

In order to determine the parameters that govern this dose response dynamics of the C_R_ and C_S_ cells, to the administration of the TMZ and Acridone derivatives, sensitivity analysis was performed. Here it was observed that the C_R_ cells were sensitive to 13 out of 29 parameter values (*p* < 0.05), while the C_S_ cells were sensitive to 12 parameters (*p* < 0.05) (Supplementary Figure 2). However, the Partial Rank Correlation Coefficient calculated for the parameters show that while the C_S_ cells are more sensitive (|PRCC| > 0.5) to the parameters governing cellular growth such as ∝_*N*_,α_*CS*_, α_*CR*_, K (carrying capacity of the tumor), and the efficacy of TMZ $\varepsilon_{\max}^{D1s}$ the parameters of C_R_ cells with |PRCC| > 0.5 comprises the dosage of Acridone derivative (D_2_), $IC_{50}^{D2r}$ and the efficacy of TMZ on the resistant cells $\varepsilon_{\max}^{D1r}$. The outcome of the analysis show that there is a strong correlation of the dosage of the Acridone derivative (D_2_) with response of the C_R_ cells which further motivates to determine the dosage combinations where synergy of the two drugs can be obtained for maximal inhibition of the drug resistant Glioma cells.

#### Drug Synergy Estimation

**Figure 9:**
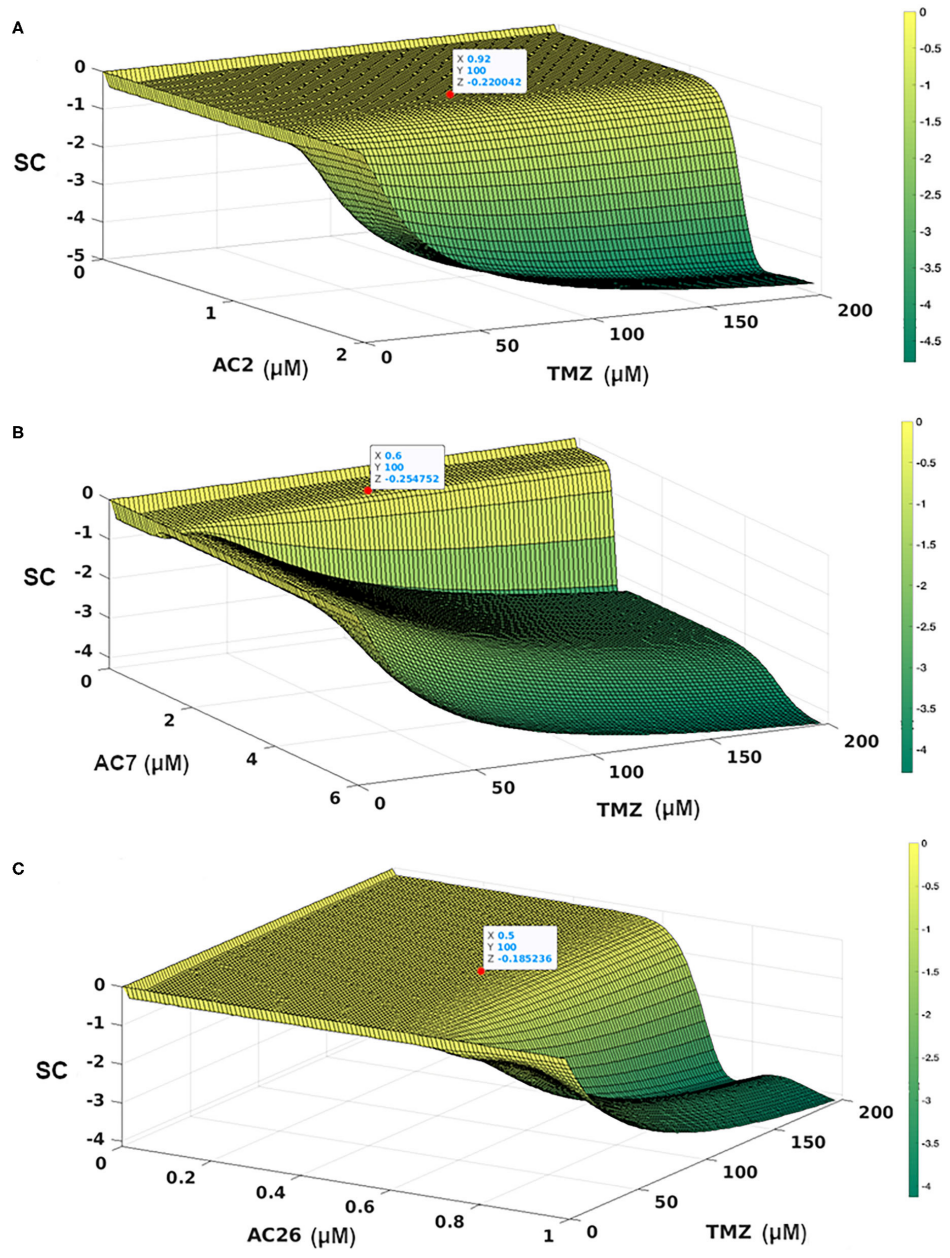
Synergy Score (SC) of 10,000 drug combinations for each of the three pairs of drugs combining the Acridone derivatives with TMZ on C_R_ cells using Bliss Independence Method. **(A)** AC2 and TMZ; **(B)** AC7 and TMZ; **(C)** AC26 and TMZ. The red points show the combinations that have been validated with experiments. The synergy scores have been calculated based on the dose-response matrix represented in Figures 8A,C,E, respectively. The color bar represents the calculated synergy scores. Here negative value represents synergy, while the positive values represent antagonism of the drug.

In order to determine the dosage combinations of the drug pairs (TMZ and Acridone derivatives) that show synergism for the drug resistant cells, the dose response matrix (Figures 8A,C,E) for the resistant cells (C_R_) for all the 10,000 dosage combinations of each of the three drug pairs (as obtained from the mathematical model) was tested for synergy. The synergy scores (SC) have been calculated based on the observed growth inhibition data obtained from the simulation (Figures 8A,C,E) with each drug pair for the resistant cell line using the Bliss Independence method. The corresponding synergy scores (SC) calculated for each combination have been shown in Figures 9A–C. Here a positive SI score shows antagonism while a negative SC score shows synergy. The 10,000 combinations were simulated for each drug pair and we observed good synergy of all the three Acridone derivatives, AC2, AC7, and AC26 when combined with TMZ for a large number of dosage combinations. The Figure 9 also shows three points on the surface plots that denote the combination that have been tested using experiment for the validation of our simulation results.

### Sulforhodamine B Assay for Combinatorial Drugs

With the leads obtained from the synergy estimations *in-silico*, CI was calculated for all the three combinatorial drug pairs using SRB assay (Table 5). The observations from the experiments showed that combinatorial drugs (TMZ+AC2, TMZ+AC7, and TMZ+AC26) showed synergistic inhibitory effect on growth of T-98 cell lines at low concentration (IC_50_) with respective CIs of 0.4, 0.41, and 0.32 (marked with red on Figures 9A–C). It has to be noted here that the Synergy Score (SC) estimated using the Bliss Independence method *in silico* (Figure 9) has been performed independently from the CI calculated from the SRB assay (Table 5). However, the result obtained from the SRB assay corroborates well within the *in-silico* findings and we observe synergy for the same drug dosage combinations from both the *in silico* and experimental studies. Our results indicate that, TMZ+AC26 shows the highest synergistic inhibitory effect amongst all the combinations.

**Table 5:** Dose-effect relationships of TMZ and AC in Glioma cell lines.

**Drugs**	**CI (T-98/TMZRES) at IC_**50**_ (μM)**
AC26 (IC_10_) + TMZ (100 μM)	0.32 ± 0.1
AC2 (IC_10_) +TMZ (100 μM)	0.4 ± 0.32
AC7 (IC_10_) + TMZ (100 μM)	0.41 ± 0.5

### Acute Toxicity

Acridone derivatives AC2, AC7, and AC26 alone and in combination with Temozolomide were evaluated for oral toxicity by administering compounds suspended in normal saline with Tween 80 through oral route. Rats were kept for overnight night fasting before the day of dosing and for 3 h after dosing. On the 1st day of dosing, they were monitored periodically for every hour upto 12 h for mortality, clinical signs, and behavioral changes. And thereafter observed twice in a day for 14 days and body weight was recorded daily. No clinical sign of toxicity was observed during the period of 14 days under observation among the control and treated groups. The gain in body weight of rats was found to be normal in the control and treated groups (Supplementary Tables 4–6). Present study suggests that acridone derivatives alone (of 2,000 mg/kg) and in combination with Temozolomide (15:1.5 mg/kg) are safe for oral administration in single dose to albino Wistar rats of female.

## Discussion

Drug resistance and recurrence are one of the major issues associated in the treatment of Glioma. Better strategies are required to be adapted for enhancing the efficacy of the treatment of Glioma patients. Development of experimentally validated mathematical models is a promising approach to estimate the efficacy of a particular therapy and predicting what percentage of tumor growth inhibition can be achieved under a particular therapy. Given the current scenario, various trial and error experiments for drug screening and drug combination study with high synergy can be carried out at economical costs and rapid rate by employing mathematical models for designing effective cancer therapy.

In this study, we propose three novel drug combinations using TMZ and Acridone derivatives as well as show their efficacy of tumor inhibition over a wide range of dosage combinations using both *in-silico* and *in-vitro* studies. The simple four variable mathematical model developed in this study captures the formation and development of a malignant Glioma and its differentiation into drug resistant and sensitive cells. This model effectively captures the cellular dynamics of a growing tumor using simple mathematical assumptions and minimal unknown parameters. The model here has been parameterized to closely mimic the behavior of U-87 drug sensitive and T-98 drug resistant Glioma cells using experimental data (Figure 6). This has been useful in screening the effectiveness and growth inhibition potential of TMZ and Acridone derivatives individually and also in combination for a wide range of doses (Figures 7, 8). Sensitivity Analysis performed on the model parameters revealed that the inhibition of the drug resistant cells correlated highly with the dosage of the Acridone derivative (D_2_) and the efficacy of TMZ on the drug resistant cells($\varepsilon_{\max}^{D1r}$) (Supplementary Figure 2). This analysis indicates a need to determine the optimum dosage of Acridone derivatives as well as the throws light on the necessity to enhance the effectiveness of the TMZ on the drug resistant cells which may be achieved through the inhibition of the resistance causing target proteins. Our analysis also reveals a significant fold change in the IC_50_ value of the drugs when used individually as opposed to when they are used in combination (Table 6). This indicates the plausibility of synergy between the drugs TMZ and Acridone derivatives. Hence, the dose response matrix obtained from the model simulations of the drug resistant cells was used to analyse the existence of synergy between TMZ and Acridone derivatives for the treatment of resistant Glioma (Figure 9). Although a key limitation of this model may be that being a deterministic model, it is governed by fixed parameters values and thus fails to capture the immense heterogeneity of Cancer cells, the effect of angiogenesis, the influence surrounding immune cells and other micro-environmental factors explicitly, it may be noted that the parameters are estimated using experimental data that has helped in calibrating the model to closely mimic a real life scenario. Nevertheless, the outcomes from our *in-silico* mathematical model corroborate well with our experimental findings and provide insights into the entire synergy landscape of these drugs when used in combination for the treatment of resistant tumors.

**Table 6:** Fold change of the combinatorial drugs.

	**Initial IC_**50**_**	**Final IC_**50**_**	**Fold change = final IC_**50**_/initial IC_**50**_**	% fold change [(final-initial)/initial]*100
**TMZ** **+** **AC26**				
TMZ	190	102.3	0.54	46.17
AC26	0.76	0.67	0.88	11.84
**TMZ** **+** **AC7**				
TMZ	190	46.2	0.24	75.68
AC7	1.05	0.96	0.91	8.5
**TMZ** **+** **AC2**				
TMZ	190	36.3	0.19	80.8
AC2	1.53	1.48	0.96	3.26

The study of drug synergy revealed that the combination of TMZ and AC26 was found to show synergistic effect on the drug resistant Glioma cell lines (T-98) as well as drug sensitive Glioma cell lines (U-87) with CI 0.6. Here we also observe that 100 μM of TMZ and IC_10_ of AC26 could effectively destroy drug sensitive cancer cells and IC_10_ of TMZ and IC_50_ of AC26 could exterminate drug resistant cancer cells. Thus, we put forward a treatment strategy i.e., use of combinatorial drugs 100 μM of TMZ and IC_10_ of AC26, which is the lowest dose possible, in order to combat drug resistance in cancers. Furthermore, we also show that these doses showing synergistic effect are below the toxicity levels shown by the individual drugs. All these results have been experimentally validated to confirm effectiveness of these combinatorial drugs.

A close observation from the results obtained, reveal a comparison between the individual activity of acridone derivatives where we note that the relative efficacy of the drugs may vary as AC26 > AC7 > AC2. These results are also confirmed from the experimental observations of drug combinations, synergy and selectivity index calculations of the drugs (Table 4) which reinforce the efficacy of the drug combinations on drug resistant cancer cells in the order TMZ + AC26 > TMZ + AC7 > TMZ + AC2. Acute toxicity studies for 14 days as per OECD guidelines have indicated that all the acridone derivatives alone and in combination with Temozolomide were found safe in single dose to female albino Wistar rats. Additionally, using *in silico* prediction tool, it has been observed that all the Acridone derivatives show BBB permeability higher than TMZ which gives hope for its clinical efficacy (Supplementary Figure 3). However, to establish the clinical usefulness of the proposed combination, further *in vitro* and *in vivo* assays are currently being undertaken for the determination of other pharmacokinetic parameters.

The molecular docking study shows that the synergistic effect of combining TMZ and Acridone derivatives might be due to inhibitory action exerted by the acridone derivatives on the resistant cells due to interactions with P-gp, MRP, and MGMT proteins that contributes to the development of drug resistance. Through the *in silico* studies it has been observed that the acridone derivatives show particularly good binding affinity to P-gp and MGMT compared to MRP. Particularly, AC26 have demonstrated highest binding affinity to all three targets P-gp, MRP, and MGMT and observed same with experimental findings. Study suggests that acridone derivatives can be further optimized for the design of safe and potent MGMT inhibitors. Good interactions at the active pocket and binding affinity of AC26 with efflux pumps and MGMT might be responsible for synergistic effect against resistant Glioma cells in combination with TMZ. Through our previous studies we have demonstrated that Acridone derivatives have DNA intercalating property which implicates that these derivatives might also be effective in killing resistant glioblastoma through MGMT-independent mechanisms as well (15, 54, 55). Whether these acridone derivatives also have an effect on the expression levels of P-gp, MRP, and MGMT proteins is currently under investigation and will be reported in another study. It is to be mentioned here that, although, in this study we have only reported the experimental verification of one dosage per drug combinations (Table 5), using our *in silico* analysis, we have been able to show the entire synergy landscape for each drug combination pair that can be tested *in vivo* using orthotropic xenografts for establishing its clinical efficacy. Along with the novel drug combinations reported in this study, we also propose a validated mathematical model, albeit simple, and *in-silico* approach to test the efficacy and synergy of novel drugs combinations in future.

## Conclusion

The novel drug combinations, involving TMZ and Acridone derivatives, proposed in this study provides new insights for the treatment of drug resistant Gliomas. The effective dosages of each of these combinations suggested in our study have been supported using both our simulation outcomes as well as experimental data. For this, the mathematical model developed here throws light on the effectiveness of each of these dosage combinations in terms of tumor reduction for wide range dosages that is not possible to screen experimentally. This is an extremely important step for the estimation of the synergistic effect of the drug pairs. Hence, it may be mentioned here that, albeit the simplicity of the model, which can be further modified in future with the inclusion of new variables, parameters and stochasticity to capture the tumor heterogeneity, the model provides useful insights in the tumor development and drug effectiveness that have been corroborated experimentally in its present form. Thus, not only does the experimental finding and docking studies of this work provide new hopes for the treatment of drug resistant Glioma, but the mathematical model developed in this study will be an invaluable tool to estimate dosage and effectiveness of other drugs for Glioma therapy in future.

## Data Availability Statement

The original contributions presented in the study are included in the article/Supplementary Material, further inquiries can be directed to the corresponding author/s.

## Ethics Statement

The animal study was reviewed and approved by The Institutional Animal Ethics Committee bearing CPCSEA/IAEC/P-52/2016 approved for undertaking the study.

## Author Contributions

MC, PG, and RS developed the mathematical model and performed the *in-silico* analysis of drug synergy. MM performed the molecular docking studies. YM and GP contributed toward experimental validation. MC, PG, and MM wrote the manuscript. RS, YM, and GP reviewed the manuscript and supervised the work. All authors contributed to the article and approved the submitted version.

## Conflict of Interest

The authors declare that the research was conducted in the absence of any commercial or financial relationships that could be construed as a potential conflict of interest.

## Supplementary Material

Supplementary Text 1 This supplementary material contains Target Proteins and Attributes of grid (Supplementary Table 1); List of parameter values (Supplementary Table 2); List of Initial Values of Model Variables (Supplementary Table 3); Toxicity Study Data of the Compound AC2 (Supplementary Table 4); Toxicity Study Data of the Compound AC7 (Supplementary Table 5); Toxicity Study Data of the Compound AC26 (Supplementary Table 6); Toxicity Study Data of Temozolomide with Compound AC2 (Supplementary Table 7); Toxicity Study Data of Temozolomide with Compound AC7 (Supplementary Table 8); Toxicity Study Data of Temozolomide with Compound AC26 (Supplementary Table 9); Histogram plots and Trace plots for Parameter Estimation using MCMC-DRAM algorithm (Supplementary Figure 1); Sensitivity Analysis of model parameters using LHS-PRCC (Supplementary Figure 2); Blood Brain Barrier Permeability results for the Acridone derivatives and TMZ (Supplementary Figure 3).

Supplementary Text 2 The codes developed for the simulation of the mathematical model have been provided. It includes the codes for (a) Parameter Estimation using the MCMC DRAM toolbox in Matlab (Scripts 1–4); (b) Determination of IC50 of TMZ and Acridone derivations from dose response curves of each drug individually in Matlab (Scripts 5 and 6); (c) Dose response matrix for each drug combinations from parameter variation studies in Matlab (Scripts 7 and 8); (d) Synergy Index calculation using Bliss Independence using SynergyFinder package in R (Script 9); (e) Sensitivity Analysis using LHS PRCC method in Matlab (Scripts 10–12).
