# Supplementary material for: Study of Combinatorial Drug Synergy of Novel Acridone Derivatives With Temozolomide Using in-silico and in-vitro Methods in the Treatment of Drug-Resistant Glioma
Source: Front Oncol. 2021 Mar 15;11:625899. doi: 10.3389/fonc.2021.625899 (PMC8006935; doi:10.3389/fonc.2021.625899)
Supplement: Supplementary Text 1 — This supplementary material contains Target Proteins and Attributes of grid (Supplementary Table 1); List of parameter values (Supplementary Table 2); List of Initial Values of Model Variables (Supplementary Table 3); Toxicity Study Data of the Compound AC2 (Supplementary Table 4); Toxicity Study Data of the Compound AC7 (Supplementary Table 5); Toxicity Study Data of the Compound AC26 (Supplementary Table 6); Toxicity Study Data of Temozolomide with Compound AC2 (Supplementary Table 7); Toxicity Study Data of Temozolomide with Compound AC7 (Supplementary Table 8); Toxicity Study Data of Temozolomide with Compound AC26 (Supplementary Table 9); Histogram plots and Trace plots for Parameter Estimation using MCMC-DRAM algorithm (Supplementary Figure 1); Sensitivity Analysis of model parameters using LHS-PRCC (Supplementary Figure 2); Blood Brain Barrier Permeability results for the Acridone derivatives and TMZ (Supplementary Figure 3). [file Data_Sheet_1.docx]

Supplementary Text 1

**Table S1: Target Proteins & Attributes of grid**

| **Name** | **Dimensions** |
| --- | --- |
| methylguanine methyltransferase enzyme (MGMT)  PDB ID- 1QNT | X 3.737838  Y 40.325753  Z 38.186650  Radius- 7.200000 |
| P-glycoprotein  PDB ID- 6QEX | X 173.330113  Y 166.742532  Z 161.482452  Radius- 7.927770 |
| multidrug resistance protein (MRP)  PDB ID- 2CBZ | X -21.272809  Y 49.011396  Z 20.419399  Radius- 8.200000 |

**Table S2: List of Parameters**

| **Sr.No.** | **Parameter** | **Values** | **Units** | **Estimates/Known** | **Ref** | **Description** |
| --- | --- | --- | --- | --- | --- | --- |
| 1 | $\propto_{N}$ | 0.4 | Hour^-1^ | Known | [1] | Rate at which cancer cells are multiplying |
| 2 | $\delta_{N}$ | 1.25 X 10^-6^ | Hour^-1^ | Known | [2] | Natural Death rate of glial cells |
| 3 | k | 1.5 X 10^6^ | Cell/ml | Estimated | [3] | Carrying capacity of cancer cells |
| 4 | $\alpha_{C}$ | 0.0233 | Hour^-1^ | Estimated | [3] | Intrinsic growth rate of cancer cells |
| 5 | $\rho$ | 0.2667 | - | Estimated | - | Transition probability of normal cells to cancer cells |
| 6 | $\delta_{C}$ | 0.092 | Hour­^-1^ | Estimated | [4] | Natural death of cancer cells |
| 7 | $\gamma_{R}$ | 0.644 | - | Expected | - | Transition Probability of Cancer cells to resistant cancer cells |
| 8 | $\omega_{S}$ | 0.356 | - | Expected | - | Transition Probability of Cancer cells to Sensitive Cancer Cells |
| 9 | $\tilde{\omega_{S}}$ | 0.034 | - | Estimated | - | Transition Probability of Cancer Resistant Cells to Cancer Sensitive Cells |
| 10 | $\tilde{\gamma_{R}}$ | 0.388 | - | Estimated | - | Transition Probability of Cancer Sensitive Cells to Cancer Resistant Cells |
| 11 | $\alpha_{CR}$ | 0.027 | Hour^-1^ | Estimated | - | Proliferation Rate of Drug Resistant Cancer Cells |
| 12 | $\delta_{R}$ | 0.0034 | Hour^-1^ | Estimated | - | Natural Death Rate of Cancer Resistant Cells |
| 13 | $\delta_{S}$ | 0.018 | Hour^-1^ | Estimated | - | Natural Death Rate of Cancer Sensitive Cells |
| 14 | $\varepsilon_{max}^{D1r}$ | 0.065 | - | Assumed based on literature evidence | [5] | Maximum effect of drug1 in drug resistant cancer cells |
| 15 | $\varepsilon_{max}^{D2r}$ | 0.11 | - | Assumed based on literature evidence | [5] | Maximum effect of drug2 in drug resistant cancer cells |
| 16 | $\varepsilon_{max}^{D3r}$ | 0.06 | - | Assumed based on literature evidence | [5] | Maximum effect of drug3 in drug resistant cancer cells |
| 17 | $\varepsilon_{max}^{D4r}$ | 0.084 | - | Assumed based on literature evidence | [5] | Maximum effect of drug4 in drug resistant cancer cells |
| 18 | $\varepsilon_{max}^{D1s}$ | 0.07 | - | Assumed based on literature evidence | [5] | Maximum effect of drug1 in drug sensitive cancer cells |
| 19 | $\varepsilon_{max}^{D2s}$ | 0.12 | - | Assumed based on literature evidence | [5] | Maximum effect of drug 2 in drug sensitive cancer cells |
| 20 | $\varepsilon_{max}^{D3s}$ | 0.069 | - | Assumed based on literature evidence | [5] | Maximum effect of drug 3 in drug sensitive cancer cells |
| 21 | $\varepsilon_{max}^{D4s}$ | 0.191 | - | Assumed based on literature evidence | [5] | Maximum effect of drug 4 in drug sensitive cancer cells |
| 22 | $\eta_{D1r}$ | 1 | - | Expected | - | Steepness of sigmoidal function for drug1 in drug resistant cancer cells |
| 23 | $\eta_{D2r}$ | 20 | - | Expected | - | Steepness of sigmoidal function for drug2 in drug resistant cancer cells |
| 24 | $\eta_{D3r}$ | 20 | - | Expected | - | Steepness of sigmoidal function for drug3 in drug resistant cancer cells |
| 25 | $\eta_{D4r}$ | 20 | - | Expected | - | Steepness of sigmoidal function for drug4 in drug resistant cancer cells |
| 26 | $\eta_{D1s}$ | 2 | - | Expected | - | Steepness of sigmoidal function for drug1 in drug sensitive cancer cells |
| 27 | $\eta_{D2s}$ | 20 | - | Expected | - | Steepness of sigmoidal function for drug2 in drug sensitive cancer cells |
| 28 | $\eta_{D3s}$ | 20 | - | Expected | - | Steepness of sigmoidal function for drug3 in drug sensitive cancer cells |
| 29 | $\eta_{D4s}$ | 14 | - | Expected | - | Steepness of sigmoidal function for drug4 in drug sensitive cancer cells |
| 30 | ${IC}_{50}^{D1s}$ | 23.33 | $\mu M$ | Known | Experimental Data | Inhibition concentration of Temozolomide in U-87 cell line |
| 31 | ${IC}_{50}^{D2s}$ | 1 | $\mu M$ | Known | Experimental Data | Inhibition concentration of Acridone 26 in U-87 cell line |
| 32 | ${IC}_{50}^{D3s}$ | 1.53 | μM | Known | Experimental Data | Inhibition concentration of Acridone 2 in U-87 cell line |
| 33 | ${IC}_{50}^{D4s}$ | 5.67 | μM | Known | Experimental Data | Inhibition concentration of Acridone 7 in U-87 cell line |
| 34 | ${IC}_{50}^{D1r}$ | 190 | $\mu M$ | Known | Experimental Data | Inhibition concentration of Temozolomide in T-98 cell line |
| 35 | ${IC}_{50}^{D2r}$ | 0.76 | $\mu M$ | Known | Experimental Data | Inhibition concentration of Acridone 26 in T-98 cell line |
| 36 | ${IC}_{50}^{D3r}$ | 1.53 | μM | Known | Experimental Data | Inhibition concentration of Acridone 2 in T-98 cell line |
| 37 | ${IC}_{50}^{D4r}$ | 1.05 | μM | Known | Experimental Data | Inhibition concentration of Acridone 7 in T-98 cell line |
| 38 | $\alpha_{CS}$ | 0.0249 | Hour^-1^ | Estimated | [6] | Proliferation rate of drug sensitive cancer cells |
| 39 | $\mu$ | 1 | Cell ml^-1^ | Known | - | Saturation constant |
| 40 | D_1_ | 0-200 | $\mu M$ | Varied | - | Dose of Temozolomide |
| 41 | D_2_ | 0-1 | $\mu M$ | Varied | - | Dose of Acridone 26 |
| 42 | D_3_ | 0-2 | μM | Varied | - | Dose of Acridone 2 |
| 43 | D_4_ | 0-6 | μM | Varied | - | Dose of Acridone 7 |

**#** Estimated parameter values have been determined using MCMC techniques. Data for the same was obtained from glioma cell lines (U-87 & T-98). Refer *Methods* of Main Article.

Assumed parameter values were found by varying the parameters within the biologically feasible ranges that were obtained from several literatures sources in order to determine its expected value for calibration of the model.

Expected parameter values were determined by varying the parameters to fit closely with the experimental observed from the SRB Assay.

**Table S3: List of Initial Values of Model Variables**

| **Sr.**  **No.** | **Variable** | **Symbol** | **Values** | **Units** | **Ref** |
| --- | --- | --- | --- | --- | --- |
| 1 | Normal Cells | N | 3000 | Cells/ml | [1] |
| 2 | Cancer Cells | C | 4951 | Cells/ml | [7] |
| 3 | Cancer Resistant Cells | $C_{R}$ | 51467 | Cells/ml | [8] |
| 4 | Cancer Sensitive Cells | $C_{S}$ | 25399 | Cells/ml | [9] |

**Results of Toxicity Studies of AC2, AC7 and AC26 in Rats for 14 days**

**Table S4: Toxicity Study Data of the Compound AC2**

| **Dose (mg/kg)** | **Body weight (g)** | | | | | | | |
| --- | --- | --- | --- | --- | --- | --- | --- | --- |
|  | Day 0 | Day 1 | Day 2 | Day 3 | Day 4 | Day 5 | Day 6 | Day 7 |
| **Control** | 165 ± 8 | 167 ± 10 | 168 ± 10 | 170 ± 8 | 171 ± 7 | 174 ± 11 | 176 ± 9 | 177 ± 10 |
| **300** | 185 ± 6 | 186 ± 12 | 189 ± 10 | 191 ± 15 | 192 ± 12 | 193 ± 22 | 195 ± 12 | 196 ± 10 |
| **2000** | 173 ± 8 | 175 ± 11 | 176 ± 8 | 178 ± 12 | 180 ± 14 | 183 ± 15 | 185 ± 12 | 188 ± 16 |

| **Dose (mg/kg)** | **Body weight (g)** | | | | | | |
| --- | --- | --- | --- | --- | --- | --- | --- |
|  | Day 8 | Day 9 | Day 10 | Day 11 | Day 12 | Day 13 | Day 14 |
| **Control** | 178 ± 8 | 181 ± 10 | 184 ± 11 | 185 ± 13 | 188 ± 8 | 190 ± 14 | 192 ± 10 |
| **300** | 194 ± 12 | 195 ± 10 | 198 ± 15 | 199 ± 16 | 201 ± 10 | 203 ± 6 | 205 ± 18 |
| **2000** | 190 ± 12 | 192 ± 16 | 195 ± 12 | 197 ± 8 | 199 ± 12 | 202 ± 15 | 206 ± 7 |

**Table S5: Toxicity Study Data of the Compound AC7**

| **Dose (mg/kg)** | **Body weight (g)** | | | | | | | |
| --- | --- | --- | --- | --- | --- | --- | --- | --- |
|  | Day 0 | Day 1 | Day 2 | Day 3 | Day 4 | Day 5 | Day 6 | Day 7 |
| **Control** | 172 ± 8 | 174 ± 12 | 175 ± 16 | 177 ± 11 | 179 ± 8 | 182 ± 10 | 184 ± 16 | 185 ± 12 |
| **300** | 175 ± 10 | 176 ± 14 | 178 ± 10 | 181 ± 8 | 183 ± 12 | 185 ± 15 | 188 ± 11 | 192 ± 12 |
| **2000** | 180 ± 8 | 181 ± 12 | 183 ± 8 | 185 ± 14 | 187 ± 6 | 189 ± 11 | 192 ± 16 | 193 ± 15 |

| **Dose (mg/kg)** | **Body weight (g)** | | | | | | |
| --- | --- | --- | --- | --- | --- | --- | --- |
|  | Day 8 | Day 9 | Day 10 | Day 11 | Day 12 | Day 13 | Day 14 |
| **Control** | 187 ± 18 | 189 ± 10 | 192 ± 12 | 194 ± 18 | 196 ± 8 | 198 ± 12 | 201 ± 16 |
| **300** | 194 ± 12 | 196 ± 14 | 195 ± 11 | 197 ± 16 | 199 ± 12 | 202 ± 10 | 205 ± 18 |
| **2000** | 197 ± 10 | 198 ± 18 | 201 ± 8 | 204 ± 10 | 206 ± 14 | 208 ± 12 | 211 ± 16 |

**Table S6: Toxicity Study Data of the Compound AC26**

| **Dose (mg/kg)** | **Body weight (g)** | | | | | | | |
| --- | --- | --- | --- | --- | --- | --- | --- | --- |
|  | Day 0 | Day 1 | Day 2 | Day 3 | Day 4 | Day 5 | Day 6 | Day 7 |
| **Control** | 185 ± 8 | 187 ± 10 | 188 ± 15 | 191 ± 12 | 194 ± 16 | 198 ± 12 | 199 ± 10 | 201 ± 12 |
| **300** | 178 ± 10 | 182 ± 8 | 183 ± 12 | 184 ± 16 | 187 ± 10 | 189 ± 16 | 191 ± 12 | 194 ± 18 |
| **2000** | 181 ± 11 | 182 ± 15 | 185 ± 10 | 188 ± 11 | 189 ± 18 | 193 ± 22 | 195 ± 16 | 199 ± 19 |

| **Dose (mg/kg)** | **Body weight (g)** | | | | | | |
| --- | --- | --- | --- | --- | --- | --- | --- |
|  | Day 8 | Day 9 | Day 10 | Day 11 | Day 12 | Day 13 | Day 14 |
| **Control** | 202 ± 18 | 204 ± 10 | 206 ± 12 | 209 ± 18 | 213 ± 11 | 215 ± 16 | 217 ± 12 |
| **300** | 196 ± 12 | 199 ± 10 | 201 ± 14 | 202 ± 16 | 205 ± 12 | 207 ± 8 | 209 ± 18 |
| **2000** | 200 ± 14 | 202 ± 10 | 206 ± 8 | 207 ± 9 | 209 ± 4 | 211 ± 12 | 215 ± 20 |

**Table S7: Toxicity Study Data of Temozolomide with Compound AC2**

| **Dose (mg/kg)**  **TMZ:AC** | **Body weight (g)** | | | | | | | |
| --- | --- | --- | --- | --- | --- | --- | --- | --- |
|  | Day 0 | Day 1 | Day 2 | Day 3 | Day 4 | Day 5 | Day 6 | Day 7 |
| **Control** | 162 ± 11 | 164 ± 14 | 165 ± 16 | 177 ± 5 | 178 ± 10 | 180 ± 14 | 181 ± 5 | 183 ± 10 |
| **10:1** | 181 ± 12 | 183 ± 8 | 185 ± 16 | 186 ± 8 | 199 ± 15 | 202 ± 18 | 205 ± 16 | 207 ± 11 |
| **15:1.5** | 192 ± 6 | 194 ± 15 | 196 ± 14 | 198 ± 10 | 201 ± 16 | 204 ± 12 | 206 ± 8 | 210 ± 12 |

| **Dose (mg/kg)**  **TMZ:AC** | **Body weight (g)** | | | | | | |
| --- | --- | --- | --- | --- | --- | --- | --- |
|  | Day 8 | Day 9 | Day 10 | Day 11 | Day 12 | Day 13 | Day 14 |
| **Control** | 184 ± 12 | 186 ± 15 | 188 ± 12 | 191 ± 11 | 193 ± 6 | 192 ± 18 | 195 ± 16 |
| **10:1** | 209 ± 14 | 211 ± 15 | 214 ± 18 | 217 ± 6 | 221 ± 14 | 225 ± 8 | 230 ± 10 |
| **15:1.5** | 212 ± 10 | 214 ± 6 | 217 ± 14 | 220 ± 16 | 224 ± 14 | 226 ± 9 | 230 ± 14 |

**Table S8: Toxicity Study Data of Temozolomide with Compound AC7**

| **Dose (mg/kg)**  **TMZ:AC** | **Body weight (g)** | | | | | | | |
| --- | --- | --- | --- | --- | --- | --- | --- | --- |
|  | Day 0 | Day 1 | Day 2 | Day 3 | Day 4 | Day 5 | Day 6 | Day 7 |
| **Control** | 185 ± 12 | 187 ± 16 | 190 ± 6 | 192 ± 14 | 195± 14 | 197 ± 16 | 200 ± 9 | 204 ± 18 |
| **10:1** | 192 ± 15 | 193 ± 10 | 195 ± 16 | 197 ± 18 | 200 ± 14 | 204 ± 12 | 207 ± 15 | 209 ± 17 |
| **15:1.5** | 165 ± 14 | 167 ± 10 | 169 ± 12 | 172 ± 18 | 175 ± 8 | 178 ± 12 | 181 ± 10 | 183 ± 14 |

| **Dose (mg/kg)**  **TMZ:AC** | **Body weight (g)** | | | | | | |
| --- | --- | --- | --- | --- | --- | --- | --- |
|  | Day 8 | Day 9 | Day 10 | Day 11 | Day 12 | Day 13 | Day 14 |
| **Control** | 206 ± 12 | 208 ± 16 | 211 ± 14 | 215 ± 16 | 218 ± 10 | 220 ± 14 | 223 ± 13 |
| **10:1** | 210 ± 8 | 213 ± 15 | 216 ± 16 | 219 ± 18 | 223 ± 16 | 225 ± 9 | 227 ± 13 |
| **15:1.5** | 185 ± 13 | 187 ± 15 | 189 ± 7 | 192 ± 13 | 195 ± 16 | 197 ± 7 | 200 ± 15 |

**Table S9: Toxicity Study Data of Temozolomide with Compound AC26**

| **Dose (mg/kg)**  **TMZ:AC** | **Body weight (g)** | | | | | | | |
| --- | --- | --- | --- | --- | --- | --- | --- | --- |
|  | Day 0 | Day 1 | Day 2 | Day 3 | Day 4 | Day 5 | Day 6 | Day 7 |
| **Control** | 179 ± 18 | 181 ± 16 | 183 ± 5 | 185 ± 15 | 188 ± 11 | 190 ± 6 | 193 ± 11 | 196 ± 15 |
| **10:1** | 167 ± 13 | 170 ± 7 | 172 ± 16 | 175 ± 11 | 178 ± 16 | 181 ± 12 | 184 ± 18 | 187 ± 13 |
| **15:1.5** | 171 ± 16 | 173 ± 18 | 175 ± 8 | 176 ± 12 | 178 ± 8 | 181 ± 17 | 184 ± 6 | 187 ± 15 |

| **Dose (mg/kg)**  **TMZ:AC** | **Body weight (g)** | | | | | | |
| --- | --- | --- | --- | --- | --- | --- | --- |
|  | Day 8 | Day 9 | Day 10 | Day 11 | Day 12 | Day 13 | Day 14 |
| **Control** | 199 ± 10 | 201 ± 16 | 204 ± 18 | 206 ± 12 | 209 ± 13 | 212 ± 12 | 215 ± 18 |
| **10:1** | 190 ± 14 | 192 ± 17 | 195 ± 18 | 197 ± 12 | 199 ± 16 | 201 ± 16 | 205 ± 12 |
| **15:1.5** | 189 ± 13 | 192 ± 15 | 195 ± 16 | 199 ± 11 | 203 ± 18 | 207 ± 14 | 210 ± 12 |


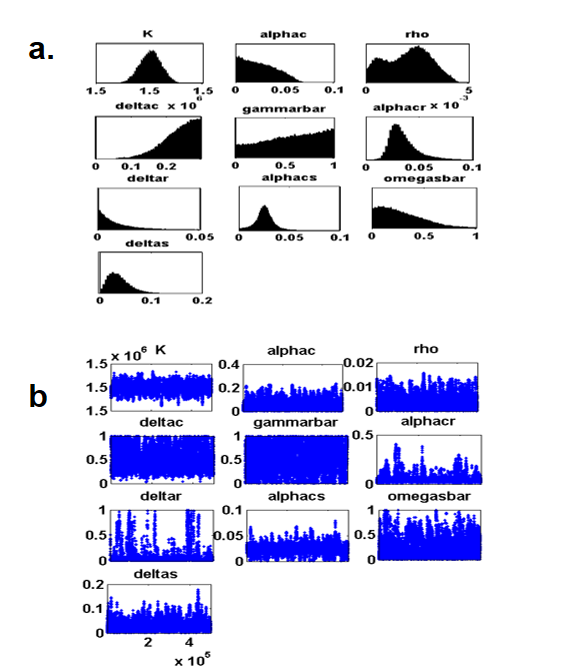


**Fig. S1**: **Histogram plots & Trace plots for Parameter Estimation using MCMC-DRAM algorithm.** (a) The histograms show posterior distribution of the estimated parameters*.* (b) Trace plots for 500,000 iterations of the estimated parameters.


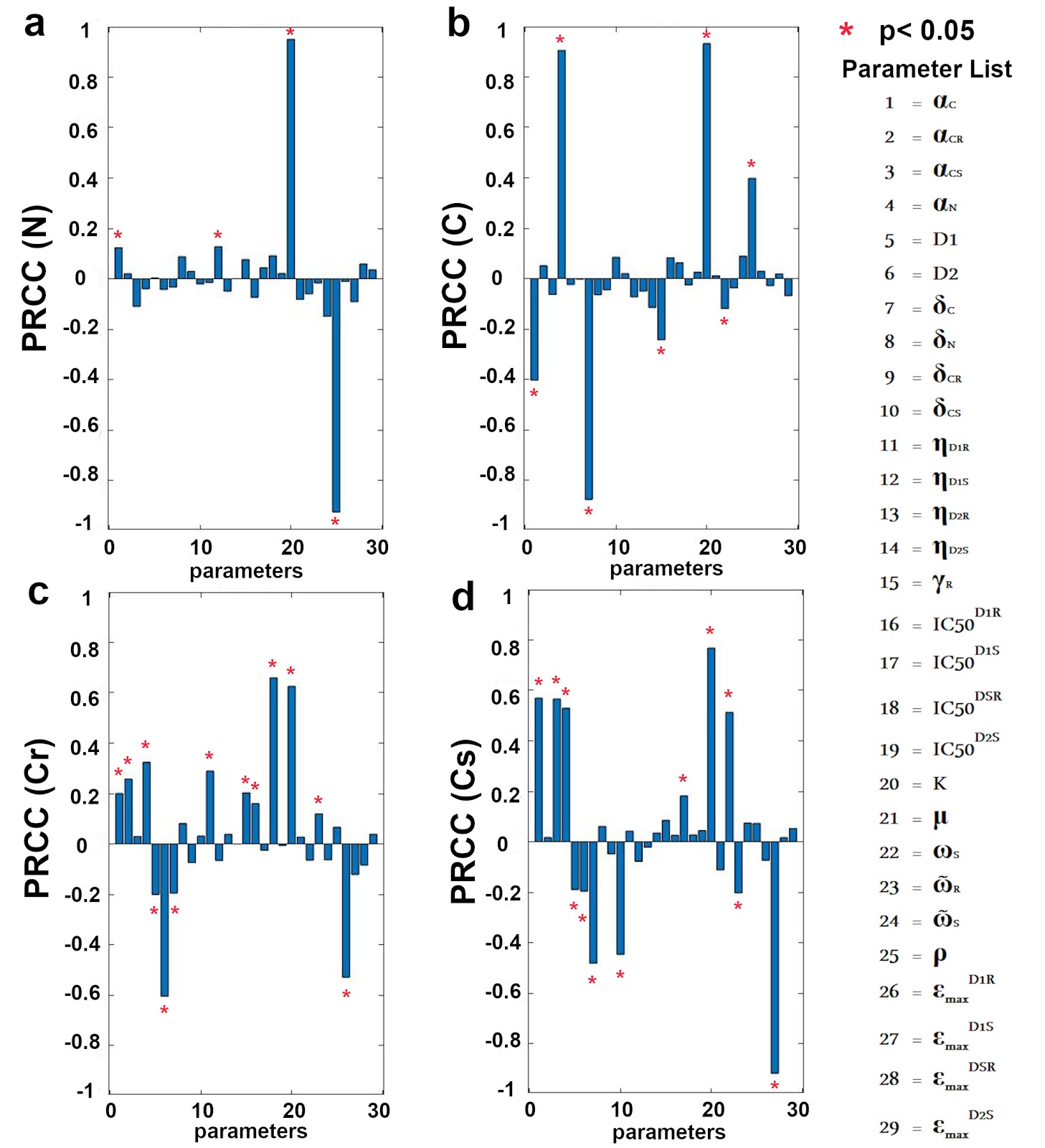


**Fig. S2: Sensitivity Analysis of model parameters using LHS-PRCC.** The sensitivity of the 29 model parameters was analysed for all the four model variables (a) Non-cancerous precursor cells (N), (b) Cancer cells (C), (c) Drug Resistant (CR) and (d) Drug sensitive (CS) cells with the effect of the TMZ and Acridone drugs. The parameters (represented on the x-axis with serial numbers) showing sensitivity (p<0.05) have been marked with red *. The PRCC values on the Y-axis represent sensitivity of the parameter based on its Partial Rank Correlation Coefficient.

**
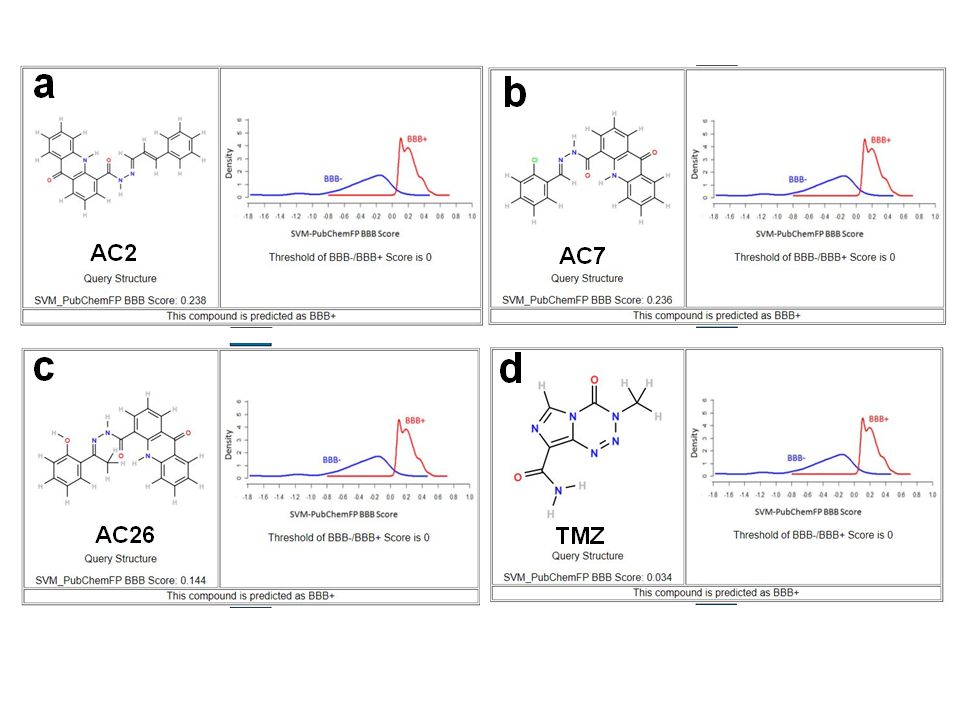
**

**Fig. S3: Blood Brain Barrier Permeability results for the Acridone derivatives and TMZ.** (a) AC2 having BBB score 0.238; (b) AC7 having BBB score 0.236 ; (c) AC26 having BBB score 0.144; (d) TMZ having BBB score 0.034

**References:**

[1] M.S. Feizabadi, T.M. Witten, Modeling drug resistance in a conjoint normal-tumor setting, Theor. Biol. Med. Model. 12 (2015) 3. https://doi.org/10.1186/1742-4682-12-3.

[2] S.I.I.I.I. Oke, A. Others, Optimal Control Analysis of a Mathematical Model for Breast Cancer, Optim. Control Appl. Methods. 37 (2018) 798–806. https://doi.org/10.1371/journal.pmed.1000097.

[3] C. Suarez, F. Maglietti, M. Colonna, K. Breitburd, G. Marshall, Mathematical Modeling of Human Glioma Growth Based on Brain Topological Structures: Study of Two Clinical Cases, PLoS One. 7 (2012) e39616. https://doi.org/10.1371/journal.pone.0039616.

[4] B.I. Camara, H. Mokrani, E. Afenya, Mathematical modeling of glioma therapy using oncolytic viruses, Math. Biosci. Eng. 10 (2013) 565–578. https://doi.org/10.3934/mbe.2013.10.565.

[5] N.F. Beggs, H.M. Dobrovolny, Determining drug efficacy parameters for mathematical models of influenza, J. Biol. Dyn. 9 (2015) 332–346. https://doi.org/10.1080/17513758.2015.1052764.

[6] X. Sun, J. Bao, Y. Shao, Mathematical Modeling of Therapy-induced Cancer Drug Resistance: Connecting Cancer Mechanisms to Population Survival Rates, Sci. Rep. 6 (2016) 22498. https://doi.org/10.1038/srep22498.

[7] B. Li, C. Fei, J. Zhang, F. Guo, A. Sun, L. Huan, S. Guo, M. Shao, Y. Jiang, Histamine induced apoptosis in primary-cultured glioma ., Biomed. Res. 28 (2017) 6725–6729.

[8] E. Castaño, P. Giménez-Bonafé, A. Tortosa, F. Martínez-Soler, J.-J. Acebes, L. Coll-Mulet, J. Gil, R. Villalonga-Planells, Activation of p53 by Nutlin-3a Induces Apoptosis and Cellular Senescence in Human Glioblastoma Multiforme, PLoS One. 6 (2011) e18588. https://doi.org/10.1371/journal.pone.0018588.

[9] D.D. Bigner, A. Winters, G.R. Choudhury, F. Yuan, R. Liu, S.T. Keir, S.-H. Yang, W. Li, L. Tang, E. Poteet, Y. Wen, H. Yan, J.W. Simpkins, A. Ghorpade, M.-G. Ryou, Reversing the Warburg Effect as a Treatment for Glioblastoma, J. Biol. Chem. 288 (2013) 9153–9164. https://doi.org/10.1074/jbc.m112.440354.
