# Supplementary material for: Study of Combinatorial Drug Synergy of Novel Acridone Derivatives With Temozolomide Using in-silico and in-vitro Methods in the Treatment of Drug-Resistant Glioma
Source: Front Oncol. 2021 Mar 15;11:625899. doi: 10.3389/fonc.2021.625899 (PMC8006935; doi:10.3389/fonc.2021.625899)
Supplement: Supplementary Text 2 — The codes developed for the simulation of the mathematical model have been provided. It includes the codes for (a) Parameter Estimation using the MCMC DRAM toolbox in Matlab (Scripts 1–4); (b) Determination of IC50 of TMZ and Acridone derivations from dose response curves of each drug individually in Matlab (Scripts 5 and 6); (c) Dose response matrix for each drug combinations from parameter variation studies in Matlab (Scripts 7 and 8); (d) Synergy Index calculation using Bliss Independence using SynergyFinder package in R (Script 9); (e) Sensitivity Analysis using LHS PRCC method in Matlab (Scripts 10–12). [file Data_Sheet_2.pdf]

SUPPLEMENTARY FOR

Study of combinatorial drug synergy of novel Acridone derivatives with Temozolomide using in-silico and in-vitro methods in the treatment of drug-resistant Glioma

M Chakravarty, P Ganguli, M Murahari, RR Sarkar, GJ Peters, and Mayur YC

CODE FOR THE SIMULATION OF THE MATHEMATICAL MODEL

The mathematical model developed for the study involves the following steps:

a) Parameter Estimation using the MCMC DRAM toolbox in Matlab (Scripts 1-4)

b) Determination of IC50 of TMZ and Acridone derivations from dose response curves of each drug individually in Matlab (Scripts 5 and 6)

c) Dose response matrix for each drug combinations from parameter variation studies in Matlab (Scripts 7 and 8)

d) Synergy Index calculation using Bliss Independence using SynergyFinder package in R (Script 9)

e) Sensitivity Analysis using LHS PRCC method in Matlab (Scripts 10-12)

===

```
%% ************************* PARAMETER ESTIMATION ********************************

%% Script 1 to 4 consists of codes written for Parameter Estimation using the package MCMCstat in Matlab
%% The package can be downloaded from the following link:
https://mjlaine.github.io/mcmcstat/#orgb3e1f3a
%% Alternatively it can be downloaded directly from
https://github.com/mjlaine/mcmcstat/archive/master.zip
```

===

```
%  Script 1:  Cancer4ex.m

clear
clc
clf
clear model data params options

% Experimental data of cell proliferation for Cancer, Cancer resistant cells & Cancer sensitive cells
data.xdata=[24;48;72;96];
data.ydata=[6026.90,100977,49085.40;6780.26,194052.8,110861.59;8878.92,346515.8,197984.96;9524.66,1029700,318121.59];
model.ssfun = @Cancer4ss;


% params = {
           % {initval minval maxval mu sigma}
          %}
params = {

        {'K',1500000, 100000, 10000000000,1500000,1}
        {'alphac',0.0014,0.001,0.8,0.0014,1}
        {'rho',0.0001,0,1,0.0001,1}
        {'deltac',0.01,0,0.3,0.01,1}%
        {'perispominegammar',0.0001,0.0001,1,0.0001,1}
        {'alphacr',0.077,0.008,0.8,0.077,1}
        {'deltar',0.000022379,0,0.1,0.000022379,1}
        {'alphacs',0.018,0.001,1,0.018,1}
        {'perispomineomegas',0.00001,0,1,0.00001,1}
        {'deltas',0.017,0.001,1,0.017,1}
       }


% model.N = length(data.ydata);  % total number of observations
% model.S20 = model.sigma2;      % prior mean for sigma2
% model.N0  = 4;                 % prior accuracy for sigma2
 model.N = [2 3 4];
 model.S20= [2 2 2];
```

```
 model.N0= [4 4 4];


% First generate an initial chain.
options.nsimu = 50000;
options.updatesigma = 4;
[results, chain, s2chain]= mcmcrun(model,data,params,options);

% % Then re-run starting from the results of the previous run,
options.nsimu = 500000;
options.updatesigma = 4;
[results, chain, s2chain] = mcmcrun(model,data,params,options,results);
%

% Chain plots should reveal that the chain has converged and we can
% use the results for estimation and predictive inference.
figure(1)
set(gca,'fontsize',16)
mcmcplot(chain,[],results,'hist');
saveas(gcf,fullfile(['mcmcHistogram4' '.fig']));

figure(2)
set(gca,'fontsize',16)
mcmcplot(chain,[],results,'denspanel',2);
saveas(gcf,fullfile(['mcmc4' '.fig']));

figure(3)
set(gca,'fontsize',16)
mcmcplot(chain,[],results);%,'pairs'
saveas(gcf,fullfile(['mcmcplot24' '.fig']));

%
% Function |chainstats| calculates mean ans std from the chain and
% estimates the Monte Carlo error of the estimates. Number |tau| is
% the integrated autocorrelation time and |geweke| is a simple test
% for a null hypothesis that the chain has converged.


chainstats(chain,results)

y0=[3000  4950.67   51466.34   25398.79];

[t,y] = ode45(@Cancer4sys,linspace(0,500),y0,[],chain(end,:));
figure
set(gca,'fontsize',16)
plot(t,y(:,1),t,y(:,2),t,y(:,3),t,y(:,4))
legend('N','C','CR','Cs')


figure
set(gca,'fontsize',16)
out = mcmcpred(results,chain,[],data.xdata,@Cancer4fun);
h = mcmcpredplot(out,data,1);

saveas(gcf,fullfile(['mcmcpredplot' '.fig']));

save MCMC_Cancer4.mat


==========================================================================================

%  Script 2:  Cancer4sys.m
%  Function call for model equations -- without drug

Function ydot =Cancer4sys(t,y,theta)
% ode system function for Cancer system

% theta($) --> parameter to be estimated

```

```
    K=theta(1);
    alphac=theta(2);
    rho=theta(3);
    deltac=theta(4);
    perispominegammar=theta(5);
    alphacr=theta(6);
    deltar=theta(7);
    alphacs=theta(8);
    perispomineomegas=theta(9);
    deltas=theta(10);
    alphan=0.4;
    deltan=1E-6;
    gammar=0.644;
    omegas=0.356;
    mu=1;


    dotN=alphan*y(1)*(1-rho)*(1-(y(1)/K))-deltan*y(1)-alphan*y(1)*rho;
    dotC=alphan*y(1)*rho + alphac*y(2)*log(K/(y(2)+
    mu))-gammar*alphac*y(2)-omegas*alphac*y(2)-deltac*y(2);%
    dotCR=gammar*alphac*y(2) + perispominegammar*alphacs*y(4) +
    alphacr*y(3)*log(K/(y(3)+ mu))-deltar*y(3)-perispomineomegas*alphacr*y(3);%

    dotCs=perispomineomegas*alphacr*y(3)+omegas*alphac*y(2)+alphacs*y(4)*log(K/(y(4)+mu))
    -deltas*y(4)-perispominegammar*alphacs*y(4);%


    ydot=[dotN;dotC;dotCR;dotCs];

=========================================================================================
%  Script 3:  Cancer4ss.m
%  Function call for sum of squares


Function ss = Cancer4ss(theta,data)

    time   = data.xdata;
    ydata  = data.ydata(:,1:end);
    %xdata  = data.xdata;

    ymodel = Cancer4fun(time,theta);
    ymodel2=ymodel(:,[2 3 4]);
    ss = sum((ymodel2- ydata).^2);

=========================================================================================
%  Script 4:  Cancer4fun.m
%  Function call for mcmc prediction

Function y=Cancer4fun(time,theta)

y0=[3000  4950.67   51466.34   25398.79];

[t,y] = ode45(@Cancer4sys,time,y0,[],theta);

=========================================================================================

%% ********************************** DOSAGE VARIATION********************************

%% Scripts 5 to 8 are for Drug Dosage Variation studies
=========================================================================================
% Script 5: param_variationTMZ.m
% Dose Response Curve (Single Drug)
% Determination of IC50 value and fitting with Relative growth observed in experimental
data using single drug
% The code for TMZ has been provided here. The same can be used for AC2, AC26 and AC7
with the correct parameter values
% as given in Table S2 of Supplementar Material

close all
```

```
clear
clc

parameter='TMZ';
param_array=[0  0.5  1  2.5  5  10  25  50  100];  %%% TMZ% Dose

n=9; %n is the no of points required between min and max of the param range
tmax=300;

for i=1:n
     param=param_array(i);

       legendinfo{i}=([parameter,'=', num2str(param)]);
        out(i,:)=ONE_ODEmodelTMZ(param,tmax,i,parameter);
         N_Growth_Percent(i,1)=(out(i,1))/out(1,1)*100;%
         C_Growth_Percent(i,1)=(out(i,2))/out(1,2)*100;%
         CR_Growth_Percent(i,1)=(out(i,3))/out(1,3)*100;%
         CS_Growth_Percent(i,1)=(out(i,4))/out(1,4)*100;%

   X(i,1)=param;
end

%Experimental data for T-98 cell line
CR_GI_exp_TMZ=[ 100; 98.25943451; 97.7437114; 96.6477998; 94.99748585; 87.42924924;
61.97831384;52.77014221;50.87234564];

figure(1)
set(gca,'FontSize',16);
semilogx(X,CR_Growth_Percent,'c','LineWidth',2)
legend('CR')

hold on
semilogx(X,CR_GI_exp_TMZ,'b','LineWidth',2)

%Experimental data for U-87 cell line
CS_GI_exp_TMZ=[ 100; 98.46943451; 97.7637114; 97; 94.92748585; 87.31924924; 50.41;
47.71; 40.87234564];

figure(2)
set(gca,'FontSize',16);
semilogx(X,CS_Growth_Percent,'c','LineWidth',2)
legend('CS')

hold on
semilogx(X,CS_GI_exp_TMZ,'b','LineWidth',2)

[rhoSTMZ,pvalSTMZ]=corr(CS_GI_exp_TMZ,CS_Growth_Percent,'Type','Spearman')
[rhoRTMZ,pvalRTMZ]=corr(CR_GI_exp_TMZ,CR_Growth_Percent,'Type','Spearman')

====================================================================================

%Script 6: ONE_ODEmodelTMZ.m
%Function Call for Dose Response Curve (Single Drug)

Function output=ONE_ODEmodelTMZ(param,tmax,i,parameter)

%estimated parameter
theta(1)=1499999.53285148;
theta(2)= 0.0233501620267144;
theta(3)= 0.266781607251610;
theta(4)= 0.0918981626307380;
theta(5)= 0.388072555621906;
theta(6)= 0.0270816495007205;
theta(7)= 0.00341313442524746;
theta(8)= 0.0249490898045103;
theta(9)= 0.0337996377364049;
theta(10)= 0.0189590390455028;
```

```
alphan=0.4;
deltan=1E-6;
K=theta(1);
alphac=theta(2);
rho=theta(3);
deltac=theta(4);
perispominegammar=theta(5);
alphacr=theta(6);
deltar=theta(7);
etaD1R=0.93;
etaD1S=1.95;
%etaD2R=20;
%etaD2S=20;
IC50D1s=25;
%IC50D2s=1;
IC50D1R=190;
%IC50D2R=0.76;
alphacs=theta(8);
gammar=0.644;
omegas=0.356;
perispomineomegas=theta(9);
deltas=theta(10);
varepsilonmaxD1R=0.064;
varepsilonmaxD1S=0.047;
varepsilonmaxD2R=0.11;
varepsilonmaxD2S=0.12;
D1=param; % TMZ
%D2=0.6;  % AC / AC7 / AC26
mu=1;

g = @(t,y)[alphan*y(1)*(1-rho)*(1-(y(1)/K))-deltan*y(1)-alphan*y(1)*rho;% normal cells%
alphan*y(1)*rho + alphac*y(2)*log(K/(y(2)+
mu))-gammar*alphac*y(2)-omegas*alphac*y(2)-deltac*y(2);% cancer cells*%
gammar*alphac*y(2) + perispominegammar*alphacs*y(4) + alphacr*y(3)*log(K/(y(3)+
mu))-deltar*y(3)-perispomineomegas*alphacr*y(3)-varepsilonmaxD1R*D1^(etaD1R)/(IC50D1R^(et
aD1R)+D1^(etaD1R))*y(3); % cancer resistant cell  %
-varepsilonmaxD2R*D2^(etaD2R)/(IC50D2R^(etaD2R)+ D2^(etaD2R))*y(3)
perispomineomegas*alphacr*y(3)+omegas*alphac*y(2)+alphacs*y(4)*log(K/(y(4)+mu))-deltas*y(
4)-perispominegammar*alphacs*y(4)-
varepsilonmaxD1S*D1^(etaD1S)/(IC50D1s^(etaD1S)+D1^(etaD1S))*y(4)]; % ];% cancer
sensitive cell % -varepsilonmaxD2S*D2^(etaD2S)/(IC50D2s^(etaD2S)+D2^(etaD2S))*y(4)
%
options = odeset('RelTol',1e-14,'AbsTol',1e-50);

[t,xa] = ode45(@(t,y) g(t,y),[0 tmax],[3000 4950.67 51466.34 25398.79]);

output=xa(end,:);

==================================================================================

% Script 7: param2_variationTMZ.m
% Dose Response Matrix (Drug Combinations)
% The code for TMZ & AC2 has been provided here. The same can be used for TMZ & AC7,
and TMZ & AC7 by substituting the correct parameter values

close all
clear
clc

%TMZ
param1Min=0;
param1Max=200;

%AC2
param2Min=0;
param2Max=2;

n=100; %n is the no of points required between min and max of the param range
tmax=300;
```

```
stepsize1=(param1Max-param1Min)/n;
stepsize2=(param2Max-param2Min)/n;

% CI_matrixM=zeros(n,n,4)
 k1=1;
 k2=1;
param2=param2Min;
    for j=1:n
        j
        param1=param1Min;
        for i=1:n

            outmatrix=TWO_ODEmodel(param1,param2,tmax); %steady state value of the
            variables for that value of param

             for var=1:4

                 out(j,i,var)=outmatrix(1,var);
                 GI_out(j,i,var)=(out(j,i,var)/out(1,1,var))*100;

             end


             X(i,1)=param1;
             param1=param1+stepsize1;
             i;
             end
   Y(j,1)=param2;
   param2=param2+stepsize2;
   j
    end
```

==

```
%Script 8: TWO_ODEmodelTMZ.m
%Function Call for Dose Response Matrix (Drug Combinations)

Function output=TWO_ODEmodel(param1,param2,tmax)%

%estimated parameter
theta(1)=1499999.53285148;
theta(2)= 0.0233501620267144;
theta(3)= 0.266781607251610;
theta(4)= 0.0918981626307380;
theta(5)= 0.388072555621906;
theta(6)= 0.0270816495007205;
theta(7)= 0.00341313442524746;
theta(8)= 0.0249490898045103;
theta(9)= 0.0337996377364049;
theta(10)= 0.0189590390455028;


alphan=0.4;
deltan=1E-6;
K=theta(1);
alphac=theta(2);
rho=theta(3);
deltac=theta(4);
perispominegammar=theta(5);
alphacr=theta(6);
deltar=theta(7);
etaD1R=1;
etaD1S=2;
etaD2R=20;
etaD2S=20;
IC50D1s=25;
IC50D2s=1.53;
IC50D1R=190;
```

```
IC50D2R=1.53;
alphacs=theta(8);
gammar=0.644;
omegas=0.356;
perispomineomegas=theta(9);
deltas=theta(10);
varepsilonmaxD1R=0.065;
varepsilonmaxD1S=0.07;
varepsilonmaxD2R=0.06;
varepsilonmaxD2S=0.069;
D1=param1;
D2=param2;
mu=1;

g = @(t,y)[alphan*y(1)*(1-rho)*(1-(y(1)/K))-deltan*y(1)-alphan*y(1)*rho;% normal cells%
alphan*y(1)*rho + alphac*y(2)*log(K/(y(2)+
mu))-gammar*alphac*y(2)-omegas*alphac*y(2)-deltac*y(2);% cancer cells*%
gammar*alphac*y(2) + perispominegammar*alphacs*y(4) + alphacr*y(3)*log(K/(y(3)+
mu))-deltar*y(3)-perispomineomegas*alphacr*y(3)-varepsilonmaxD1R*D1^(etaD1R)/(IC50D1R^(et
aD1R)+D1^(etaD1R))*y(3)-varepsilonmaxD2R*D2^(etaD2R)/(IC50D2R^(etaD2R)+
D2^(etaD2R))*y(3);% cancer resistant cell  %
perispomineomegas*alphacr*y(3)+omegas*alphac*y(2)+alphacs*y(4)*log(K/(y(4)+mu))-deltas*y(
4)-perispominegammar*alphacs*y(4)-
varepsilonmaxD1S*D1^(etaD1S)/(IC50D1s^(etaD1S)+D1^(etaD1S))*y(4)-varepsilonmaxD2S*D2^(eta
D2S)/(IC50D2s^(etaD2S)+D2^(etaD2S))*y(4)]; % ];% cancer sensitive cell %

options = odeset('RelTol',1e-5,'AbsTol',1e-16);
[t,xa] = ode45(@(t,y) g(t,y),[0 tmax],[3000 4950.67 51466.34 25398.79],options);

output=xa(end,:);
```

===

```
% ******************************** SYNERGY INDEX ***********************************

% SynergyFinder package can be downloaded and installed in R studio
% Input: Dose Response Matrix for Drug Combinations as obtained from the simulation
```

===

```
% Script 9: SynFin.R
library(synergyfinder)
library(readxl)
dataset <- read_excel("S:/Malobika/2020/inputfiles13012020/AC2_TMZ.xlsx")
View(dataset)

syn_vals <- vector()
for (i in 1:3)
{
  x_seed=floor(runif(1, min=0, max=10000))
  set.seed(x_seed)
  dose.response.mat <- ReshapeData(dataset, data.type = "viability",impute = TRUE,
  noise = TRUE,correction = "all??")
  #str(dose.response.mat)
  synergy.score <- CalculateSynergy(data = dose.response.mat,method = "Bliss")
  file_name<-paste('AC2_results_',i,'.csv',sep='')
  write.csv(synergy.score[["scores"]], file=file_name)
  xmat <- read.csv(file_name, header = FALSE, sep= ",", skip=0)
  xmat <- as.matrix(xmat)
  syn_vals[i] <- as.numeric(xmat[97,98])
}
write.csv(syn_vals, file='syn_val.csv')

% The plots for the snergy indices were made using the heatmap function in Matlab
```

===

```
% ****************************** SENSITIVITY ANALYSIS *****************************
% Sensitivity Analysis using the LHS-PRCC package in Matlab
% The package is available from the following link:
```

http://malthus.micro.med.umich.edu/lab/usadata/

```
% Scripts 10-13 are required for the Sensitivity Analysis after downloading the
LHS-PRCC package

==================================================================================


% Script 10: Model_LHS.m

clear
clc
close all

%% Sample size N
runs=300;

%% LHS MATRIX  %%
Parameter_settings_LHS;

alphac_LHS=LHS_Call(0.00233501620267144,    alphac, 0.233501620267144,
0.00233501620267144,runs,'norm');
alphacr_LHS=LHS_Call(0.00270816495007205, alphacr, 0.270816495007205,
0.00270816495007205, runs, 'norm');
alphacs_LHS=LHS_Call(0.00249490898045103, alphacs, 0.249490898045103,
0.00249490898045103, runs, 'norm');
alphan_LHS=LHS_Call(0.04, alphan, 4, 0.04, runs, 'norm');
D1_LHS=LHS_Call(10, D1, 1000, 10, runs, 'norm');
D2_LHS=LHS_Call(0.06, D2, 6, 0.06, runs, 'norm');
deltac_LHS=LHS_Call(0.0091898162630738, deltac, 0.91898162630738, 0.0091898162630738,
runs, 'norm');
deltan_LHS=LHS_Call(0.0000001, deltan, 0.00001, 0.0000001, runs, 'norm');
deltar_LHS=LHS_Call(0.000341313442524746, deltar, 0.0341313442524746,
0.000341313442524746, runs, 'norm');
deltas_LHS=LHS_Call(0.00189590390455028, deltas, 0.189590390455028,
0.00189590390455028, runs, 'norm');
etaD1R_LHS=LHS_Call(0.093, etaD1R, 9.3, 0.093, runs, 'norm');
etaD1S_LHS=LHS_Call(0.195, etaD1S, 19.5, 0.195, runs, 'norm');
etaD2R_LHS=LHS_Call(2, etaD2R, 200, 2, runs, 'norm');
etaD2S_LHS=LHS_Call(2, etaD2S, 200, 2, runs, 'norm');
gammar_LHS=LHS_Call(0.0644, gammar, 1, 0.0644, runs, 'norm');
IC50D1R_LHS=LHS_Call(19, IC50D1R, 1900, 19, runs, 'norm');
IC50D1s_LHS=LHS_Call(2.5, IC50D1s, 250, 2.5, runs, 'norm');
IC50D2R_LHS=LHS_Call(0.076, IC50D2R, 7.6, 0.076, runs, 'norm');
IC50D2s_LHS=LHS_Call(0.1, IC50D2s, 10, 0.1, runs, 'norm');
K_LHS=LHS_Call(149999.953285148, K, 14999995.3285148, 149999.953285148, runs, 'norm');
mu_LHS=LHS_Call(0.1, mu, 10, 0.1, runs, 'norm');
omegas_LHS=LHS_Call(0.0356, omegas, 1, 0.0356, runs, 'norm');
perispominegammar_LHS=LHS_Call(0.0388072555621906, perispominegammar, 1,
0.0388072555621906, runs, 'norm');
perispomineomegas_LHS=LHS_Call(0.00337996377364049, perispomineomegas, 1,
0.00337996377364049, runs, 'norm');
rho_LHS=LHS_Call(0.026678160725161, rho, 1, 0.026678160725161, runs, 'norm');
varepsilonmaxD1R_LHS=LHS_Call(0, varepsilonmaxD1R, 1, 0.01, runs, 'norm');
varepsilonmaxD1S_LHS=LHS_Call(0, varepsilonmaxD1S, 1, 0.01, runs, 'norm');
varepsilonmaxD2R_LHS=LHS_Call(0, varepsilonmaxD2R, 1, 0.01, runs, 'norm');
varepsilonmaxD2S_LHS=LHS_Call(0, varepsilonmaxD2S, 1, 0.01, runs, 'norm');


%% LHS MATRIX and PARAMETER LABELS
LHSmatrix=[alphac_LHS   alphacr_LHS alphacs_LHS alphan_LHS  D1_LHS  D2_LHS  deltac_LHS
deltan_LHS  deltar_LHS  deltas_LHS  etaD1R_LHS  etaD1S_LHS  etaD2R_LHS  etaD2S_LHS
gammar_LHS  IC50D1R_LHS  IC50D1s_LHS     IC50D2R_LHS    IC50D2s_LHS  K_LHS  mu_LHS
omegas_LHS  perispominegammar_LHS   perispomineomegas_LHS   rho_LHS
varepsilonmaxD1R_LHS    varepsilonmaxD1S_LHS    varepsilonmaxD2R_LHS
varepsilonmaxD2S_LHS];

figure
for x=1:runs %Run solution x times choosing different values
    f=@ODE_LHS;
    x
```

```
    LHSmatrix(x,:);
    [t,y]=ode15s(@(t,y)f(t,y,LHSmatrix,x,runs),tspan,y0,[]);
    Anew=[t y]; % [time y]
    %% Save the outputs at ALL time points [tspan]

    N_lhs(:,x)=Anew(:,2);
    C_lhs(:,x)=Anew(:,3);
    Cr_lhs(:,x)=Anew(:,4);
    Cs_lhs(:,x)=Anew(:,5);

end

% CALCULATE PRCC
PRCC_var={'alphac_LHS', 'alphacr_LHS',  'alphacs_LHS',  'alphan_LHS',   'D1_LHS',
'D2_LHS',   'deltac_LHS',   'deltan_LHS',   'deltar_LHS',   'deltas_LHS',
'etaD1R_LHS',   'etaD1S_LHS',   'etaD2R_LHS',   'etaD2S_LHS',   'gammar_LHS',
'IC50D1R_LHS',  'IC50D1s_LHS',  'IC50D2R_LHS',  'IC50D2s_LHS',  'K_LHS',    'mu_LHS',
'omegas_LHS',   'perispominegammar_LHS',    'perispomineomegas_LHS',    'rho_LHS',
'varepsilonmaxD1R_LHS', 'varepsilonmaxD1S_LHS', 'varepsilonmaxD2R_LHS',
'varepsilonmaxD2S_LHS'};

figure
[prcc_N sign sign_label]=PRCC(LHSmatrix,N_lhs,1:300,PRCC_var,0.01,'N');
figure

[prcc_C sign sign_label]=PRCC(LHSmatrix,C_lhs,1:300,PRCC_var,0.01,'C');
figure

title('C')
[prcc_Cr sign sign_label]=PRCC(LHSmatrix,Cr_lhs,1:300,PRCC_var,0.01,'Cr');
figure

title('Cr')
[prcc_Cs sign sign_label]=PRCC(LHSmatrix,Cs_lhs,1:300,PRCC_var,0.01,'Cs');
figure

PRCC_PLOT(LHSmatrix,N_lhs,300,PRCC_var,'prcc_N');
PRCC_PLOT(LHSmatrix,C_lhs,300,PRCC_var,'prcc_C');
PRCC_PLOT(LHSmatrix,Cr_lhs,300,PRCC_var,'prcc_Cr');
PRCC_PLOT(LHSmatrix,Cs_lhs,300,PRCC_var,'prcc_Cs');

save SensitivityAnalysis_Model_LHS.mat;

=========================================================================================
% Script 11: ODE_LHS.m
% Function call for Sensitivity Analysis

Function dydt=ODE_LHS(t,y,LHSmatrix,x,runs)
% % PARAMETERS % %
Parameter_settings_LHS;

alphac=LHSmatrix(x,1);
alphacr=LHSmatrix(x,2);
alphacs=LHSmatrix(x,3);
alphan=LHSmatrix(x,4);
D1=LHSmatrix(x,5);
D2=LHSmatrix(x,6);
deltac=LHSmatrix(x,7);
deltan=LHSmatrix(x,8);
deltar=LHSmatrix(x,9);
deltas=LHSmatrix(x,10);
etaD1R=LHSmatrix(x,11);
etaD1S=LHSmatrix(x,12);
etaD2R=LHSmatrix(x,13);
etaD2S=LHSmatrix(x,14);
gammar=LHSmatrix(x,15);
IC50D1R=LHSmatrix(x,16);
IC50D1s=LHSmatrix(x,17);
IC50D2R=LHSmatrix(x,18);
```

```
IC50D2s=LHSmatrix(x,19);
K=LHSmatrix(x,20);
mu=LHSmatrix(x,21);
omegas=LHSmatrix(x,22);
perispominegammar=LHSmatrix(x,23);
perispomineomegas=LHSmatrix(x,24);
rho=LHSmatrix(x,25);
varepsilonmaxD1R=LHSmatrix(x,26);
varepsilonmaxD1S=LHSmatrix(x,27);
varepsilonmaxD2R=LHSmatrix(x,28);
varepsilonmaxD2S=LHSmatrix(x,29);



dotN=alphan*y(1)*(1-rho)*(1-(y(1)/K))-deltan*y(1)-alphan*y(1)*rho;% normal cells%

dotC=alphan*y(1)*rho + alphac*y(2)*log(K/(y(2)+
mu))-gammar*alphac*y(2)-omegas*alphac*y(2)-deltac*y(2);% cancer cells*%

dotCr=gammar*alphac*y(2) + perispominegammar*alphacs*y(4) + alphacr*y(3)*log(K/(y(3)+
mu))-deltar*y(3)-perispomineomegas*alphacr*y(3)-varepsilonmaxD1R*D1^(etaD1R)/(IC50D1R^(et
aD1R)+D1^(etaD1R))*y(3)-varepsilonmaxD2R*D2^(etaD2R)/(IC50D2R^(etaD2R)+
D2^(etaD2R))*y(3);% cancer resistant cell  %

dotCs=perispomineomegas*alphacr*y(3)+omegas*alphac*y(2)+alphacs*y(4)*log(K/(y(4)+mu))-del
tas*y(4)-perispominegammar*alphacs*y(4)-
varepsilonmaxD1S*D1^(etaD1S)/(IC50D1s^(etaD1S)+D1^(etaD1S))*y(4)-varepsilonmaxD2S*D2^(eta
D2S)/(IC50D2s^(etaD2S)+D2^(etaD2S))*y(4); %

dydt = [dotN;dotC;dotCr;dotCs];

==================================================================================
% Script 12: Parameter_settings_LHS.m
% Function call for Sensitivity Analysis


% PARAMETER BASELINE VALUES
alphac=0.0233501620267144;
alphacr=0.0270816495007205;
alphacs=0.0249490898045103;
alphan=0.4;
D1=100;
D2=0.6;
deltac=0.0918981626307380;
deltan=1E-6;
deltar=0.00341313442524746;
deltas=0.0189590390455028;
etaD1R=0.93;
etaD1S=1.95;
etaD2R=20;
etaD2S=20;
gammar=0.644;
IC50D1R=190;
IC50D1s=25;
IC50D2R=0.76;
IC50D2s=1;
K=1499999.53285148;
mu=1;
omegas=0.356;
perispominegammar=0.388072555621906;
perispomineomegas=0.0337996377364049;
rho=0.266781607251610;
varepsilonmaxD1R=0.064;
varepsilonmaxD1S=0.047;
varepsilonmaxD2R=0.11;
varepsilonmaxD2S=0.12;


% Parameter Labels
```

```
PRCC_var={'alphac_LHS' , 'alphacr_LHS','alphacs_LHS' , 'alphan_LHS','D1_LHS' , 'D2_LHS'
, 'deltac_LHS' , 'deltan_LHS' , 'deltar_LHS' , 'deltas_LHS' , 'etaD1R_LHS' ,
'etaD1S_LHS' , 'etaD2R_LHS' , 'etaD2S_LHS' , 'gammar_LHS' , 'IC50D1R_LHS' ,
'IC50D1s_LHS' , 'IC50D2R_LHS' , 'IC50D2s_LHS' , 'K_LHS' , 'mu_LHS' , 'omegas_LHS' ,
'perispominegammar_LHS' , 'perispomineomegas_LHS' , 'rho_LHS' , 'varepsilonmaxD1R_LHS'
, 'varepsilonmaxD1S_LHS' , 'varepsilonmaxD2R_LHS' , 'varepsilonmaxD2S_LHS'};

%% TIME SPAN OF THE SIMULATION
t_end=10000; % length of the simulations
tspan=(1:1:t_end);   % time points where the output is calculated
time_points=[50 300]; % time points of interest for the US analysis

% INITIAL CONDITION FOR THE ODE MODEL

y0=[3000 4950.67 51466.34 25398.79];

% Variables Labels
y_var_label={'N','C','Cr','Cs'};
```
